# *Geosmithia* Species Associated With Bark Beetles From China, With the Description of Nine New Species

**DOI:** 10.3389/fmicb.2022.820402

**Published:** 2022-03-14

**Authors:** Xiuyu Zhang, You Li, Hongli Si, Guoyan Zhao, Miroslav Kolařík, Jiri Hulcr, Xiaoqian Jiang, Meixue Dai, Runlei Chang

**Affiliations:** ^1^College of Life Sciences, Shandong Normal University, Jinan, China; ^2^Vector-Borne Virus Research Center, Fujian Province Key Laboratory of Plant Virology, Fujian Agriculture and Forestry University, Fuzhou, China; ^3^College of Plant Protection, Fujian Agriculture and Forestry University, Fuzhou, China; ^4^School of Forests, Fisheries and Geomatics Sciences, University of Florida, Gainesville, FL, United States; ^5^Institute of Microbiology, Czech Academy of Sciences, Prague, Czechia

**Keywords:** fungal community, symbiosis, 9 new taxa, *Geosmithia*, bark beetles

## Abstract

Fungi of the genus *Geosmithia* are frequently associated with bark beetles that feed on phloem on various woody hosts. Most studies on *Geosmithia* were carried out in North and South America and Europe, with only two species being reported from Taiwan, China. This study aimed to investigate the diversity of *Geosmithia* species in China. Field surveys in Fujian, Guangdong, Guangxi, Hunan, Jiangsu, Jiangxi, Shandong, Shanghai, and Yunnan yielded a total of 178 *Geosmithia* isolates from 12 beetle species. The isolates were grouped based on morphology. The internal transcribed spacer, β-tubulin, and elongation factor 1-α gene regions of the representatives of each group were sequenced. Phylogenetic trees were constructed based on those sequences. In total, 12 species were identified, with three previously described species (*Geosmithia xerotolerans*, *G. putterillii*, and *G. pallida*) and nine new species which are described in this paper as *G. luteobrunnea*, *G. radiata*, *G. brevistipitata*, *G. bombycina*, *G. granulata* (*Geosmithia* sp. 20), *G. subfulva*, *G. pulverea* (*G.* sp. 3 and *Geosmithia* sp. 23), *G. fusca*, and *G. pumila* sp. nov. The dominant species obtained in this study were *G. luteobrunnea* and *G. pulverea*. This study systematically studied the *Geosmithia* species in China and made an important contribution to filling in the gaps in our understanding of global *Geosmithia* species diversity.

## Introduction

Members of *Geosmithia* are widely distributed fungal associates of phloem- and xylem-feeding beetles (Pitt, 1979; Kolařík et al., 2007, 2017; Lin et al., 2016), such as species in Bostrichidae and Curculionidae-Scolytinae (Coleoptera) (Juzwik et al., 2015; Kolařík et al., 2017). *Geosmithia* species are predominantly isolated from phloem-feeding bark beetles on broadleaved and conifer trees although they have been documented from many other substrates including soil (Kolařík et al., 2004), seed-feeding beetles (Huang et al., 2017), animal skin (Crous et al., 2018), indoor environment (Crous et al., 2018), insect-free plant tissues (McPherson et al., 2013), and food materials (Pitt and Hocking, 2009). To date, almost 60 phylogenetic and 21 formally described *Geosmithia* species have been recognized (Strzałka et al., 2021).

*Geosmithia* is similar to *Penicillium* and *Paecilomyces* in morphology, but it can be distinguished by the combination of stipe with or without a curved basal cell, verrucose conidiophores (including phialide), cylindrical phialide shape with a very short and cylindrical neck (collula), and ellipsoidal or cylindrical conidia (except globose conidia in *Geosmithia eupagioceri* and *G. microcorthyli*). The colony color could be in shades of white, yellow, brown, or red but never bluish-green or green (Kolařík et al., 2004; Kolařík and Kirkendall, 2010).

The spores of *Geosmithia* may be transmitted by attaching to the surface of the beetle vector, but the ecological role of most *Geosmithia* species in symbiosis with bark beetles is still unclear. Some species serve as the main food source or supplementary nutrition for the beetles (Kolařík and Kirkendall, 2010; Machingambi et al., 2014), but most are probably commensals with minimal or no benefit to the beetle (Veselská et al., 2019) because the vector beetles show neither any apparent morphological adaptation nor nutrient dependence (Huang et al., 2017, 2019). Some *Geosmithia* species exhibit extracellular antimicrobial and antifungal metabolites, but their ecological implications are unknown (Stodůlková et al., 2009; Veselská et al., 2019).

Some *Geosmithia* species can cause serious tree diseases. One example is the thousand cankers disease (TCD) of walnuts caused by *G. morbida* (Kolařík et al., 2011). Following high-density colonization by its beetle vector, the walnut twig beetle (*Pityophthorus juglandis*), in the phloem of walnut (*Juglans* spp.) or wingnut (*Pterocarya* spp.) trees, *G. morbida* causes numerous small lesions which eventually girdle the vascular tissue (Tisserat et al., 2009; Kolařík et al., 2011; Utley et al., 2012; Seybold et al., 2013; Hishinuma et al., 2015). TCD has affected many walnut trees in North America, especially in the western United States (Tisserat et al., 2009, 2011), and has recently been detected in Europe (Montecchio et al., 2014). Another mildly pathogenic species *Geosmithia* sp. 41 causes mild pathogenicity in *Quercus argifolia* (Kolařík et al., 2017), originally reported as *G. pallida* (Lynch et al., 2014).

After the discovery of the *Geosmithia*–beetle association (Kirschner, 2001), there has been an accumulation of reports describing *Geosmithia* fungi from phloem-feeding bark beetles around the world (Kolařík et al., 2004, 2007, 2008; Kubátová et al., 2004; Kolarik et al., 2005; Kolařík and Jankowiak, 2013; McPherson et al., 2013; Jankowiak et al., 2014; Machingambi et al., 2014; Pepori et al., 2015; Huang et al., 2019; Strzałka et al., 2021). Fungal communities associated with phloem-infected bark beetles are formed by a variety of biological and abiotic factors. The tree host is one of the most important selection factors (Skelton et al., 2018). Like other beetle-vectored fungi such as the ophiostomatoid fungi (Seifert et al., 2013), *Geosmithia* species display variable degrees of specificity to their beetle vectors and tree hosts, ranging from generalists to single-species specialists (Kolařík et al., 2007, 2008; Kolařík and Jankowiak, 2013; Jankowiak et al., 2014; Veselská et al., 2019). Other factors affecting the fungal community structure include beetle ecology, surrounding host tree community, and climatic factors (Six and Bentz, 2007; Jankowiak et al., 2014). These factors also influence the communities of *Geosmithia*, most notably by the fact that different beetles infesting the same host tree have similar *Geosmithia* assemblages (Kolařík et al., 2008; Machingambi et al., 2014).

At present, most of the studies of *Geosmithia* were conducted in North and South America and Europe, but the mycoflora of Asian bark beetles remain understudied. This study investigated the *Geosmithia* species from China using phylogenetic analyses and morphological and physiological features, and nine *Geosmithia* new species are described to fill the gap in our understanding of the global *Geosmithia* diversity.

## Materials and Methods

### Sampling, Isolating, and Preserving Fungal Isolates

The beetle gallery samples were collected in Fujian, Guangdong, Guangxi, Hunan, Jiangsu, Jiangxi, Shandong, Shanghai, and Yunnan Province from plant hosts of *Altingia gracilipes* (Altingiaceae), *Gnetum luofuense* (Gnetaceae), Lauraceae sp., *Liquidambar formosana* (Altingiaceae), *L. styraciflua* (Altingiaceae), *Eriobotrya japonica* (Rosaceae), *Acacia pennata* (Mimosaceae), *Rhus chinensis* (Anacardiaceae), *Cupressus funebris* (Cupressaceae), and *Ulmus* spp. (Ulmaceae) and kept individually in sealable bags (Table 1). The adult beetles were individually placed in Eppendorf tubes. Both galleries and adult beetles were kept at 4°C for further isolation. The beetle vectors included three groups: (1) Curculionidae-Scolytinae: *Acanthotomicus suncei*, *Scolytus jiulianshanensis* (Curculionidae-Scolytinae), *S. semenovi*, *Microperus* sp. L589, *Cryphalus eriobotryae*, *C. kyotoensis*, and *Phloeosinus* sp. and *P.* cf. *hopehi*, (2) Curculionidae-Platypodinae: *Crossotarsus emancipates*, and (3) Bostrichidae: *Dinoderus* sp. L489, *Sinoxylon* cf. *cucumellae* and *Xylocis tortilicornis* (Table 1). The fungal isolates were obtained by scraping wood tissue from the beetle galleries and crushing the beetle coating. The isolates were inoculated on 2% malt extract agar [MEA: 20 g agar (Solarbio, China), 20 g malt extract (Hopebio, China), and 1 L deionized water] amended with 0.05% streptomycin. The cultures were purified by hyphal-tip subculturing and incubated at 25°C. All the cultures obtained in this study were deposited in the culture collection (SNM) of Shandong Normal University, Jinan, Shandong Province, China. Isolates linked to type specimens of the fungal species were deposited in the China General Microbiological Culture Collection Center (CGMCC), Beijing, China. The holotype specimens (dry cultures) were deposited in the Herbarium Mycologicum, Academiae Sinicae (HMAS), Beijing, China (Table 2).

**TABLE 1:** Distribution and number of species of *Geosmithia* among 178 isolated strains.

*Geosmithia* species	Location	Tree host	Beetle species	Beetle groups	Gallery/beetle	No.
*G. bombycina* (2)	Fujian	*Eriobotrya japonica*	*Cryphalus eriobotryae*	Curculionidae-Scolytinae	Gallery	2
*G. brevistipitata* (18)	Shandong	*Cupressus funebris*	*Phloeosinus* cf. *hopehi*	Curculionidae-Scolytinae	Gallery	18
*G. fusca* (26)	Yunnan	*Acacia pennata*	*Sinoxylon* cf. *cucumella*	Bostrichidae	Beetle	8
	Guangdong	*Phyllanthus emblica*	*Xylocis tortilicornis*	Bostrichidae	Gallery	10
		*Hibiscus tiliaceus*	*Ernoporus japonicus*	Curculionidae-Scolytinae	Gallery	8
*G. granulata* (30)	Yunnan	*Acacia pennata*	*Sinoxylon* cf. *cucumella*	Bostrichidae	Beetle	2
	Guangdong	*Hibiscus tiliaceus*	*Ernoporus japonicus*	Curculionidae-Scolytinae	Gallery	26
	Jiangsu	*Ulmus* sp.	*Scolytus semenovi*	Curculionidae-Scolytinae	Gallery	2
*G. luteobrunnea* (39)	Jiangxi	*Liquidambar formosana*	*Acanthotomicus suncei*	Curculionidae-Scolytinae	Gallery	25
					Beetle	1
		*Ulmus* sp.	*Scolytus jiulianshanensis*	Curculionidae-Scolytinae	Gallery	5
	Shanghai	*Liquidambar styraciflua*	*Acanthotomicus suncei*	Curculionidae-Scolytinae	Gallery	8
*G. pallida* (2)	Yunnan	*Acacia pennata*	*Sinoxylon* cf. *cucumella*	Bostrichidae	Gallery	2
*G. pulverea* (33)	Guangdong	*Gnetum luofuense*	*Dinoderus* sp.	Bostrichidae	Gallery	1
	Shanghai	*Liquidambar styraciflua*	*Acanthotomicus suncei*	Curculionidae-Scolytinae	Gallery	1
	Yunnan	*Acacia pennata*	*Sinoxylon* cf. *cucumella*	Bostrichidae	Beetles	8
	Guangxi	Unknown	*Crossotarsus emancipatus*	Curculionidae-Platypodinae	Gallery	2
	Hunan	Unknown	*Microperus* sp. L589	Curculionidae-Scolytinae	Gallery	1
	Fujian	*Eriobotrya japonica*	*Cryphalus eriobotryae*	Curculionidae-Scolytinae	Gallery	1
	Shandong	*Rhus chinensis*	*Cryphalus kyotoensis*	Curculionidae-Scolytinae	Gallery	1
					Beetle	2
	Jiangsu	*Ulmus* sp.	*Scolytus semenovi*	Curculionidae-Scolytinae	Gallery	4
	Jiangxi	*Liquidambar formosana*	*Acanthotomicus suncei*	Curculionidae-Scolytinae	Gallery	1
		Unknown	*Phloeosinus* sp.	Curculionidae-Scolytinae	Gallery	6
		*Ulmus* sp.	*Scolytus jiulianshanensis*	Curculionidae-Scolytinae	Beetle	1
		*Altingia gracilipes*	*Acanthotomicus suncei*	Curculionidae-Scolytinae	Gallery	4
*G. pumila* (2)	Jiangsu	*Ulmus* sp.	*Scolytus semenovi*	Curculionidae-Scolytinae	Gallery	2
*G. putterillii* (6)	Jiangxi	*Lauraceae*	*Phloeosinus* sp.		Gallery	6
*G. radiata* (14)	Jiangxi	*Liquidambar formosana*	*Acanthotomicus suncei*	Curculionidae-Scolytinae	Gallery	7
		*Ulmus* sp.	*Scolytus jiulianshanensis*	Curculionidae-Scolytinae	Gallery	1
		*Altingia gracilipes*	*Acanthotomicus suncei*	Curculionidae-Scolytinae	Gallery	6
*G. subfulva* (5)	Guangdong	*Hibiscus tiliaceus*	*Ernoporus japonicus*	Curculionidae-Scolytinae	Beetle	2
	Fujian	*Rhus chinensis*	*Hypothenemus* sp. L636	Curculionidae-Scolytinae	Beetle	2
	Shandong	*Rhus chinensis*	*Cryphalus kyotoensis*	Curculionidae-Scolytinae	Gallery	1
*G. xerotolerans* (1)	Shandong	*Cupressus funebris*	*Phloeosinus* cf. *hopehi*	Curculionidae-Scolytinae	Gallery	1

**TABLE 2:** Cultures examined in this study and their GenBank accession numbers.

				GenBank accession no.				
Species	Isolation no.	Beetle vectors	Tree host	ITS	TEF1-α	TUB2	RPB2	References
*G. bombycine*	***SNM934***	*Cryphalus eriobotryae*	*Eriobotrya japonica*	MZ519396	MZ514871	MZ514862	OL825679	Present study
	***SNM933^T^* = *CGMCC3.20578***	*C. eriobotryae*	*E. japonica*	MZ519395	MZ514870	MZ514861	OL825678	Present study
*G. brevistipitata*	***SNM1616^T^* = *CGMCC3.20627***	*Phloeosinus* cf. *hopehi*	*Cupressus funebris*	OK584392	OK632357	OK632375	OL825675	Present study
	***SNM1610***	*Phloeosinus* cf. *hopehi*	*C. funebris*	OK584393	OK632356	OK632373	OL825677	Present study
	***SNM1611***	*Phloeosinus* cf. *hopehi*	*Cupressus funebris*	OK584394	OK632355	OK632374	OL825676	Present study
*G. brunnea*	CBS 142634	*Xylosandrus compactus*	*Liquidambar styraciflua*	KY872741	KY872746	KY872751	KY882266	Huang et al., 2017
	CBS 142635	*X. compactus*	*L. styraciflua*	KY872742	KY872747	KY872752	KY882268	Huang et al., 2017
	CBS 142633*^T^*	*Hypothenemus dissimilis*	*Quercus* sp.	KY872743	KY872748	KY872753	KY882268	Huang et al., 2017
*G. cnesini*	CCF 3753	*Cnesinus lecontei*	*Croton draco*	AM947670				Kolařík and Kirkendall, 2010
	MK 1820	*C. lecontei*	*C. draco*	AM947671				Kolařík and Kirkendall, 2010
*G. eupagioceri*	MKA1-b	*Eupagiocerus dentipes*	*Paullinia renesii*	AM947666				Kolařík and Kirkendall, 2010
	CCF 3754				LR535705		LR535704	Kolarík et al., 2019*
*G. fagi*	CCF 6235	*Taphrorychus bicolor*	*Fagus sylvatica*	LR812775	LR813193	LR813119		Strzałka et al., 2021
	21114TBb	*T. bicolor*	*F. sylvatica*	LR812776		LR813120		Strzałka et al., 2021
	CCF 6234^T^	*T. bicolor*	*F. sylvatica*	LR812785	LR813141	LR813129		Strzałka et al., 2021
*G. fassatiae*	AK 31/98	*S. intricatus*	*Quercus* sp.	AM421039	MH580557			Kolařík et al., 2008
	CCF 4331			HF546239		KF853894		Kolařík et al., 2012*
	CCF 4340			HF546247		KF853895		Kolařík et al., 2012*
	CCF 3334 *^T^*		*Quercus pubescens*		MH580530			Kolarik et al., 2005
*G. flava*	CCF 3333^T^	*Xiphydria* sp.	*Castanea sativa*	AJ578483	MH580541			Kolařík et al., 2004
	CCF4337	Cerambycidae sp.	*Pseudotsuga menziesii*	HF546244	MH580542	KF853897		Kolařík et al., 2004
	CCF3354						LR535685	Kolarík et al., 2019*
*G. fusca*	***SNM1577***	*Phyllanthus emblica*	*Xylocis tortilicornis*	OK584387	OK632359	OK632371	OL825662	Present study
	***SNM1578^T^* = *CGMCC3.20626***	*Phy. Emblica*	*Xylocis tortilicornis*	OK584388	OK632358	OK632370	OL825661	Present study
	***SNM1012* = *CGMCC3.20486***	*Sinoxylon* cf. *cucumella*	*Acacia pennata*	MZ519390	MZ514866	MZ514857	OL825664	Present study
	***SNM1167***	*Sin.* cf. *cucumella*	*Aca. pennata*	MZ519392	MZ514865	MZ514856	OL825663	Present study
*G. granulate*	***SNM1015^T^* = *CGMCC3.20450***	*Sin.* cf. *cucumella*	*Aca. pennata*	MZ519398	MZ514873	MZ514864	OL825667	Present study
	***SNM1013***	*Sin.* cf. *cucumella*	*Aca. pennata*	MZ519397	MZ514872	MZ514863	OL825668	Present study
*G. lavendulan*	CCF 3051		Laboratory contamination	AF033385				Kolařík et al., 2004
	CCF 3394	*Carphoborus vestitus*	*Pistacia terebinthus*	AM421098				Kolařík et al., 2007
	Hulcr 17347				MH580484			Present study
	CCF 4336					KF853902		Hamelin et al., 2013
*G. langdonii*	CCF 3332*^T^*	*Scolytus intricatus*	*Quercus robur*	KF808297	HG799876	HG799887	HG799928	Kolarik et al., 2005; Kolařík et al., 2017
	CCF 4338	*C. pubescens*	*Sequoia serpervirens*	HF546245	HG799877	HG799881	HG799929	Kolařík et al., 2017
*G. longistipitata*	RJ278m	*Pityophthorus pityographus*	*Picea abies*	HE604124				Strzałka et al., 2021
	CCF 4210^T^	*P. pityographus*	*P. abies*	HE604154	LR813194	LR813140		Strzałka et al., 2021
*G. luteobrunnea*	***SNM261^T^* = *CGMCC3.20252***	*S. jiulianshanensis*	*Ulmus* sp.	MW222399	MW592410	MW592395	OL825669	Present study
	***SNM226***	*A. suncei*	*L. styraciflua*	MW222404	MW592426	MW592392	OL825670	Present study
	***SNM287***	*A. suncei*	*L. styraciflua*	MW222393	MW592406	MW592398	OL825671	Present study
	***SNM256* = *CGMCC3.20254***	*A. suncei*	*L. formosana*	MW222401	MW592423	MW592403	OL825674	Present study
*G. microcorthyli*	CCF 3861 *^T^*	*Microcorthylus* sp.	*Cassia grandis*	FM986798	MH580560	FM986793	FM986794	Kolařík and Kirkendall, 2010
*G. morbida*	CBS 124664		*Juglans nigra*	FN434081		KF853912	LR535706	Kolařík et al., 2011
	CCF 3881	*Pityophthorus juglandis*	*J. nigra*	FN434082	MH580543	KF853911		Kolařík and Kirkendall, 2010
	CCF 4576	*P. juglandis*	*J. nigra*		MH580544			Kolařík et al., 2007
*G. obscura*	CCF 3422^T^	*S. intricatus*	*Q. robur*	AJ784999	MH580539			Kolarik et al., 2005
	CCF 3425	*S. carpini*	*Carpinus betulus*	AM181460	MH580540	KF853914		Kolarik et al., 2005
*G. omnicola*	MK 1707	*Hylesinus orni*	*Fraxinus* sp.	AM181452	MH580558			Kolařík et al., 2008
	CNR115		*Ulmus minor*			KP990607		Pepori et al., 2015
	CNR5		*Ulmus glabra*			KP990546		Pepori et al., 2015
	IMI 194089	*H. orni*	*Fraxinus* sp.	AM181450				Kolařík et al., 2008
	CCF 3553	*H. orni*	*Fraxinus* sp.	AM181433				Kolařík et al., 2008
*G. pallida*	CCF 3053*^T^*		*Cotton yarn*	AJ578486		HG799817	HG799908	Kolařík et al., 2004, 2017
	CCF 3324		Soil		HG799846	HG799809	HG799900	Kolařík et al., 2004, 2017
	**SNM1165**	*Sin.* cf. *cucumella*	*Aca. pennata*	MZ519393	MZ514868	MZ514859	OL825666	Present study
	**SNM1166**	*Sin.* cf. *cucumella*	*Aca. pennata*	MZ519394	MZ514869	MZ514860	OL825665	Present study
*G. pazoutovae*	22015aSI	*S. intricatus*	*Q. robur*	LR812786		LR813130		Strzałka et al., 2021
	24Wa14SI	*S. intricatus*	*Q. robur*	LR812787		LR813131		Strzałka et al., 2021
	CCF 6233^T^	*S. intricatus*	*Q. robur*	LR812796	LR813142	LR813138		Strzałka et al., 2021
*G. proliferans*	CBS 142636*^T^*	*Phloeotribus frontalis*	*Acer negundo*	KY872744	KY872749	KY872754	KY882269	Huang et al., 2017
	CBS 142637	*P. frontalis*	*A. negundo*	KY872745	KY872750	KY872755	KY882270	Huang et al., 2017
*G. pulverea*	***SNM885^T^* = *CGMCC3.20255***	*Dinoderus* sp.	*Gnetum luofuense*	MW222410	MW592415	MW592388	OL825656	Present study
	***SNM270***	*A. suncei*	*L. formosana*	MW222398	MW592421	MW592387	OL825659	Present study
	***SNM248***	*A. suncei*	*L. styraciflua*	MW222402	MW592424	MW592386	OL825657	Present study
	**SNM886**	*Crossotarsus emancipatus*		MW222411	MW592416	MW592385	OL825658	Present study
	**SNM887**	*C. emancipatus*		MW222412	MW592417	MW592384	OL825660	Present study
	**SNM888**	*Microperus* sp.	*Choerospondias axillaris*	MW222409	MW592414	MW592389	OL825655	Present study
*G. pumila*	***SNM1653^T^* = *CGMCC3.20630***		*Ulmus pumila*	OK584389	OK632361	OK632366	OL825653	Present study
	***SNM1657***		*Ulmus pumila*	OK584390	OK632360	OK632367	OL825654	Present study
*G. putterillii*	CCF 3052*^T^*		*Beilschmiedia tawa*	AF033384	HG799853	HG799816	HG799907	Kolařík et al., 2004, 2017
	U 307		*B. tawa*	HF546306	MH580529			Kolařík et al., 2004, 2017
	**SNM402**	*Phloeosinus* sp.		MW584874	MW592419	MW592405	-	Present study
	**SNM436**	*Phloeosinus* sp.		MW584873	MW592418	MW592404	-	Present study
*G. radiata*	***SNM279^T^* = *CGMCC3.20253***	*A. suncei*	*L. formosana*	MW222397	MW592420	MW592402	OL825672	Present study
	***SNM884***	*A. suncei*	*L. formosana*	MW222406	MW592411	MW592400	OL825673	Present study
*G. rufescens*	MK 1800	*C. lecontei*	*C. draco*	AM947667				Kolařík and Kirkendall, 2010
	MK 1803	*C. lecontei*	*C. draco*	AM947668			LR535708	Kolařík and Kirkendall, 2010
	MK 1821	*C. lecontei*	*C. draco*	AM947669		KF853927		Kolařík and Kirkendall, 2010
	CCF 3752				LR535709			Kolarík et al., 2019*
*G. subfulva*	***SNM1304^T^* = *CGMCC3.20579***	*Hibiscus tiliaceus*	*Ernoporus japonicus*	OK584385	OK632363	OK632368	OL825651	Present study
	***SNM1298***	*H. tiliaceus*	*Ernoporus japonicus*	OK584386	OK632362	OK632369	OL825652	Present study
*G. ulmacea*	CCF 3559	*S. multistriatus*	*Ulmus* sp.	AM181439	MH580535			Kolařík et al., 2008
	1226	*S. schevyrewi*	*Ulmus* sp.	KJ716463				Zerillo et al., 2014*
	CNR23		*U. minor*			KP990560		Pepori et al., 2015
	CNR24		*U. minor*			KP990561		Pepori et al., 2015
*G. xerotolerans*	CCF 5270	*Scolytus oregoni*	*P. menziesii*		MH580534			Kolařík et al., 2017
	FMR 17085*^T^*			NR_169923		LS998791		
	CCF 4280	*H. ficus*	*F. carica*	AM421049	MH580533			Kolařík et al., 2007
	**SNM1618**	*Phloeosinus* cf. *hopehi*	*Cupressus funebris*	OK584391	OK632354	OK632372	-	Present study
	CCF4334					KF853939		Hamelin et al., 2013
*Geosmithia* sp. 2	U107	*Scolytus rugulosus*	*Prunus* sp.	HF546256	HG799855	HG799818	HG799910	Kolařík et al., 2017
	MK 642	*H. orni*	*Fraxinus ornus*		HG799852		HG799906	Kolařík et al., 2017
*Geosmithia* sp. 3	CCF 4298	*S. intricatus*	*Quercus dalechampii*	AM181436	HG799851	HG799814	HG799905	Kolařík et al., 2008, 2017
	CCF 3481	*Scolytus carpini*	*C. betulus*	AM181467	HG799842	HG799805	HG799896	Kolařík et al., 2017
*Geosmithia* sp. 4	CCF 4278	*Pteleobius vittatus*	*Ulmus laevis*	AM181466	HG799850	HG799813	HG799904	Kolařík et al., 2008, 2017
*Geosmithia* sp. 5	CCF 3341	*S. intricatus*	*Quercus petraea*	AJ578487	HG799837	HG799801	HG799891	Kolařík et al., 2004, 2017
	CCF 4215	*P. pityographus*	*P. abies*	HE604117			HG799909	Kolařík and Jankowiak, 2013
	AK192/98	*S. intricatus*	*Q. robur*		HG799835		HG799889	Kolařík et al., 2017
*Geosmithia* sp. 8	CCF 3358	*S. intricatus*	*Q. petraea*	AM181421	MH580559	FM986788		Kolařík and Kirkendall, 2010
*Geosmithia* sp. 9	CCF 3564			AM181428				Kolařík et al., 2008
	CCF 3702			AM746018				Kolařík and Jankowiak, 2010*
	RJ0266	*Ips cembrae*	*Larix decidua*		MH580551			Kolařík and Jankowiak, 2013
*Geosmithia* sp. 11	CCF 3555	*S. intricatus*	*Q. pubescens*	AM181419	MH580545	KF853931		Kolařík et al., 2008
	CCF 3556	*S. intricatus*	*Q. pubescens*	AM181418				Kolařík et al., 2008
*Geosmithia* sp. 12	CCF 4320	*Hylesinus oregonus*	*Fraxinus*sp.	HF546229	MH580532	KF853932		Kolařík et al., 2017
	CCF 3557	*Leperisinus orni*	*F. excelsior*	AM181431	MH580531			Kolařík et al., 2008
*Geosmithia* sp. 16	CCF 4201	*P. pityographus*	*P. abies*	HE604146	HE604206	HE604181	HE604234	Kolařík and Jankowiak, 2013
	RJ34m	*P. pityographus*	*P. abies*			HE604182	HE604259	Kolařík and Jankowiak, 2013
*Geosmithia* sp. 19	CCF 3658	*Hypoborus ficus*	*Ficus carica*	AM421085	MH580546			Kolařík et al., 2007
	CCF 3655	*H. ficus*	*F. carica*	AM421075				Kolařík et al., 2007
*Geosmithia* sp. 20	CCF 4316	*Phloesinus fulgens*	*Calocedrus decurrens*	HF546226	MH580547			Kolařík et al., 2017
	U193	*Scolytus schevyrewi*	*Ulmus pumila*	HF546287	MH580548			Kolařík et al., 2017
*Geosmithia* sp. 22	CCF 3645	*Phloetribus scarabeoides*	*Olea europaea*	AM421061	MH580552	KF853941		Kolařík et al., 2007
	CCF 3652	*P. scarabeoides*	*O. europaea*	AM421062	MH580553			Kolařík et al., 2007
*Geosmithia* sp. 23	CCF 3318	*Scolytid beetles*	*Persea gratissima*	AJ578489		HG799808	HG799899	Kolařík et al., 2004, 2017
	CCF 3639	*Scolytus rugulosus*	*Prunus armeniaca*	AM421068	HG799838	HG799802	HG799892	Kolařík et al., 2004, 2017
	U160	*Scolytus multistriatus*	*U. pumila*	HF546284			HG799911	Kolařík et al., 2017
*Geosmithia* sp. 24	MB136	*Orthotomicus erosus*	*Pinus halepensis*	KP691926		KP691936		Dori-Bachash et al., 2015
	MB242	*Pityogenes calcaratus*	*Pinus brutia*	KP691927		KP691937		Dori-Bachash et al., 2015
	MB322	*O. erosus*	*P. brutia*	KP691928		KP691938		Dori-Bachash et al., 2015
	CCF 4294	*Pityogenes quadridens*	*P. sylvestris*		MH580555			Kolařík and Jankowiak, 2013
	MK1772	*P. pityographus*	*P. sylvestris*		MH580556			Kolařík and Jankowiak, 2013
*Geosmithia* sp. 25	MK1832	*Cryphalus abietis*	*Abies alba*	HE604128	HE604218	HE604186	HE604250	Kolařík and Jankowiak (2013)
	CCF 4205	*Cryphalus piceae*	*A. alba*	HE604127	HE604219	HE604187	HE604253	Kolařík and Jankowiak, 2013
*Geosmithia* sp. 26	CCF 4222	*Pinus sylvestris*		HE604158	LN907595			Kolařík et al., 2017
*Geosmithia* sp. 27	CCF 4206	*Pityogenes bidentatus*	*P. sylvestris*	HE794978	HG799839		HG799893	Kolařík et al., 2017
	CCF 4605	*Pityophthorus* sp.	*Pinus ponderosae*	HF546309		HG799827	HG799919	Kolařík and Jankowiak, 2013
*Geosmithia* sp. 29	CCF 4221	*C. piceae*	*A. alba*	HE604125	HE604233	HE604184	HE604248	Kolařík and Jankowiak, 2013
*Geosmithia* sp. 30	CCF 4288	*I. cembrae*	*L. decidua*	HE604132	HE604216	HE604193	HE604242	Kolařík and Jankowiak, 2013
*Geosmithia* sp. 31	CCF 4196	*P. pityographus*	*P. sylvestris*		HE604230	HE604176	HE604256	Kolařík and Jankowiak, 2013
*Geosmithia* sp. 32	CCF 3554	*Phloeosinus thujae*	*Chamaecyparis pisifera*	AM181426	HG799874	HG799885	HG799926	Kolařík et al., 2008, 2017
	CCF 5242	*Phloeosinus sequiae*	*S. serpervirens*	HF546265	HG799873	HG799886	HG799925	Kolařík et al., 2008, 2017
*Geosmithia* sp. 33	CCF 4598	*Scolytus praeceps*	*Abies concolor*	HF546331	HG799869	HG799831	HG799923	Kolařík et al., 2017
*Geosmithia* sp. 34	CCF 4604	*Ips plastographus*	*C. decurrens*	HF546295	HG799866	HG799826	HG799918	Kolařík et al., 2017
	U417	*S. praeceps*	*A. concolor*	HF546330	HG799868	HG799830	HG799922	Kolařík et al., 2017
*Geosmithia* sp. 35.	U196	*Pityophthorus* sp	*P. menziesii*	HF546231		HG799823		Kolařík et al., 2017
*Geosmithia* sp. 36	CCF 4328	*Pityophthorus* sp.	*Pinus muricata*	HF546236				Kolařík et al., 2017
	MK1814		*Cedrus atlantica*		MH580538			Present study
*Geosmithia* sp. 37	U197	*Pityophthorus* sp.	*P. menziesii*	HF546288	HG799862	HG799824	HG799915	Kolařík et al., 2017
*Geosmithia* sp. 38	U79	*Pseudopityophthorus pubipennis*	*Notholithocarpus densiflorus*	HF546346	MH580537			Kolařík et al., 2017
	CCF 5241	*P. pubipennis*	*Quercus acrifolia*	HF546251	MH580536			Kolařík et al., 2017
*Geosmithia* sp. 39	U323	*P juglandis*	*Juglans hindsii*	HF546314		KC222335		Kolařík et al., 2017
*Geosmithia* sp. 40	CCF 5250	*Pityophthorus* sp.	*Pinus ponderosa*	HF546273	MH580550			Kolařík et al., 2017
	CCF 5245	*I. plastographus*	*Pinus radiata*	HF546304	MH580549			Kolařík et al., 2017
*Geosmithia* sp. 41	U215	*Cossoninae* sp.	*Artemisia arborea*	HF546292	HG799865	HG799825	HG799917	Kolařík et al., 2017
	CCF 4342	*Bostrichidae* sp.	*Toxicodendron diversilobum*	HF546249	HG799871	HG799833	HG799924	Kolařík et al., 2017
	U64	*Scobicia declivis*	*Umbellularia californica*	HF546342	HG799870	HG799832	HG799930	Kolařík et al., 2017
*Geosmithia* sp. 42	U166	*P. canadensis*	*Chamaecyparis* sp.	HF546279	HG799860	HG799821	HG799912	Kolařík et al., 2017
	CCF 5251	*S. rugulosus*	*Prunus* sp.	HF546285	HG799861	HG799822	HG799913	Kolařík et al., 2017
*Geosmithia* sp. 43	CCF 4203	*Pityogenes knechteli*	*P. ponderosae*	HF546223	HG799864		HG799916	Kolařík et al., 2017
*Geosmithia* sp. 44	CCF 4333	*Pityophthorus* sp.	*Pinus sabiniana*		LN907598			Kolařík et al., 2017
	CCF 4332	*Pityophthorus* sp.	*P. sabiniana*		LN907599			Kolařík et al., 2017
*Geosmithia* sp. 45	Hulcr 17004	*Pityophthorus annectens*	*Pinus taeda*		MH580482			Huang et al., 2019
	Hulcr 17006	*P. annectens*	*P. taeda*		MH580487			Huang et al., 2019
	Hulcr 18823	*Pityophthorus pulicarius*	*P. taeda*		MH580505			Huang et al., 2019
*Geosmithia* sp. 46	Hulcr 11575	*Pseudopityophthorus minutissimus*	*Quercus laurifolia*	MH426748	MH580479			Huang et al., 2019
	Hulcr 18077	*Hypothenemus eruditus*	*J. nigra*	MH426766	MH580490			Huang et al., 2019
	Hulcr 18201	*H. eruditus*	*J. nigra*	MH426776	MH580501			Huang et al., 2019
*Geosmithia* sp. 47	Hulcr 11904	*H. dissimilis*	*Q. laurifolia*	MH426749	MH580480			Huang et al., 2019
	Hulcr 19182	*H. dissimilis*	*Carya illinoinensis*	MH426789	MH580510			Huang et al., 2019
*Geosmithia* sp. 48	Hulcr 19190	*Phloeosinus dentatus*	*Juniperus virginiana*	MH426796	MH580514			Huang et al., 2019
	Hulcr 19192	*P. dentatus*	*J. virginiana*	MH426797	MH580515			Huang et al., 2019
*Emericellopsis pallida*	CBS 490.71	*Pityophthorus* sp.		NR_145052	KC998998	KC987138	KC999034	Grum-Grzhimaylo et al., 2013

The isolates recovered in the present study are in bold. Emericellopsis pallida was selected as the outgroup of phylogenies. Strains in italics were screened for morphological studies. *The sequences are available on NCBI but have not been published.

### DNA Extraction, Amplification, and Sequencing

DNA was extracted by scraping fresh fungal tissue from pure cultures and adding to 50 μl extraction solution of the PrepMan Ultra Sample Preparation Reagent (Applied Biosystems, Foster City, CA, United States). The samples were vortexed for 1 min, incubated at 100°C for 10 min, and then centrifuged at 5,000 rpm (MiniSpin Plus Centrifuge, Eppendorf 5453, Germany) for 5 min. The supernatant was transferred to a new Eppendorf tube and used as the template for polymerase chain reaction (PCR) amplification.

The rDNA region of ITS1-5.8S-ITS2, internal transcribed spacer (ITS), was amplified using the primer pair of ITS1-F (Gardes and Bruns, 1993) and ITS4 (White et al., 1990). The translation elongation factor 1-α gene (TEF1-α) was amplified using the primer pair of EF1-983F and EF1-2218R (Rehner and Buckley, 2005). β-Tubulin (TUB2) was amplified by using T10 and Bt2b (Glass and Donaldson, 1995; O’Donnell and Cigelnik, 1997). The second-largest subunit of the RNA polymerase II gene (RPB2) was amplified using the primer pair of fRPB2-5F/fRPB2-7cR (Liu et al., 1999). The PCR amplifications were carried out in a final 25-μl PCR reaction mixture consisting of 50–100 ng template DNA, 1.25 U Taq polymerase (Vazyme Biotech Co., Ltd., Nanjing, China), 200 μM dNTP, 0.5 μM of each primer, and 5% (v/v) dimethyl sulfoxide. The PCR conditions were as follows: 95°C for 3 min, followed by 30 cycles of 95°C for 1 min, 55°C for 1 min, and 72°C for 1 min. The final extension step was 72°C for 10 min. The amplified products were sequenced in Sangon Biotech, Qingdao, Shandong Province, China.

### DNA Sequence Analyses

The sequences obtained using the forward and reverse primers were aligned in Geneious version 10.2.2 (Biomatters, Auckland, New Zealand). The reference sequences of *Geosmithia* species were retrieved from GenBank (Table 2). *Emericellopsis pallida* CBS 490.71 was chosen as the phylogenetic outgroup. The sequences were aligned by using the online version of MAFFT v. 7 (Katoh and Standley, 2013) with the default settings. The best nucleotide substitution model for each partition was determined in jModelTest v. 2.1.1 (Darriba et al., 2012). Maximum parsimony (MP) analyses were performed using MEGA v.10.2.0 with 1,000 bootstrap replicates; gaps were treated as a fifth-state character. Maximum likelihood (ML) phylogenetic analyses were conducted in the CIPRES Science Gateway (Miller et al., 2010) using RAxML v. 8.2.2 (Stamatakis, 2014) with the recommended partition parameters to assess the tree topology and bootstrap values from 1,000 replicate searches. Bayesian inference (BI) was estimated in the CIPRES Science Gateway (Miller et al., 2010) using MrBayes 3.2.7a (Ronquist et al., 2012). The MCMC runs of four chains were executed simultaneously from a random starting tree for 20 million generations, and every 100 generations were sampled, resulting in 200,000 trees. Chain convergence was determined with Tracer 1.7^1^, and the effective sample size values over 200 are considered adequate. A total of 50,000 trees were discarded during burn-in. Posterior probabilities were estimated from the retained 150,000 trees. Phylogenetic trees were visualized and edited in FigTree v. 1.4.3. The final alignments used in this study have been submitted to TreeBase^2^ (nos.: 28242).

### Morphological Study

Morphological characters were observed and recorded using Olympus BX61 microscope (Olympus Corporation, Tokyo, Japan). The images were analyzed using ImageJ^3^. At least 50 measurements for each of the structures were measured. The results of the calculation are expressed as (minimum -) mean minus standard deviation -- mean plus standard deviation -- (- maximum). One-way ANOVA in SPSS version 26.0^4^ was used to evaluate the morphological differences of the different species, with a significance level of 0.05 (Supplementary Figure S5).

### Growth Study

Three independently isolated strains of each novel taxon were randomly selected for the growth experiments. The actively growing edge mycelia were inoculated at the center of 90-mm Petri dishes containing 2% MEA and incubated in darkness at temperatures ranging from 5 to 35°C at 5°C intervals and 37°C for 8 days; each temperature had three duplicates. The colony diameters were measured every 2 days, and then the optimum temperature of growth for each species and the high- and low-temperature conditions of growth were calculated.

## Results

In total, 125 samples (*N*) were collected (Table 1). A total of 178 strains in the genus *Geosmithia* were isolated from 12 beetle species and their galleries. One hundred fifty-eight strains were from the galleries and 20 strains were from the beetles. There were 63 strains from Jiangxi, 47 from Guangdong, 23 from Shandong, 20 from Yunnan, 9 from Shanghai, 8 from Jiangsu, 5 from Fujian, 2 from Guangxi, and 1 from Hunan (Table 1).

### Phylogenetic Analysis

**FIGURE 1:**
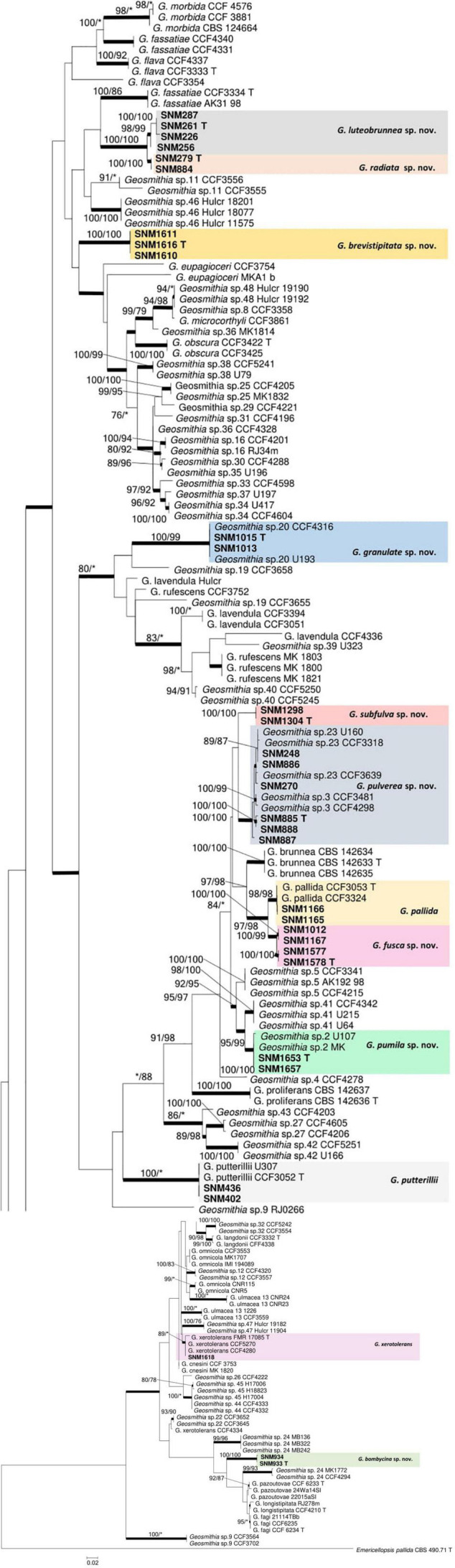
Maximum likelihood (ML) tree of *Geosmithia* generated from the combined ITS, TEF1-α, TUB2, and RPB2 sequence data. The sequences generated from this study are printed in bold. The bold branches indicate posterior probability values ≥0.9. Bootstrap values of ML/maximum parsimony ≥75% are recorded at the nodes. T, ex-type isolates.

The preliminary classification was carried out by BLAST on NCBI GenBank using the ITS marker (Supplementary Table S1). Subsequently, 32 representative strains were selected for multi-gene phylogenetic analysis, and 24 strains were screened for morphological studies (Table 2). The aligned sequences, including gaps, yielded 555 characters for ITS where 124 were parsimony informative, 899 characters for TEF1-α where 209 were parsimony informative, 1,066 characters for RPB2 where 380 were parsimony informative, and 653 characters for TUB2 where 321 were parsimony informative. The concatenated dataset comprised 162 sequences covering 3,173 characters where 1,028 were parsimony informative. The final average standard deviation of split frequency of MCMC analysis was 0.009591 for the concatenated dataset, 0.004862 for ITS, 0.006573 for TEF1-α, 0.008026 for RPB2, and 0.007595 for TUB2. The best substitution model for ITS, TEF1-α, RPB2, TUB2, and combined alignment was GTR + I + G. For all datasets (ITS, TUB2, TEF1-α, and RPB2), ML, MP, and Bayesian inference produced nearly identical topologies, with slight variations in the statistical support for each of the individual sequence datasets (Figure 1 and Supplementary Figures S1–S4). Phylograms obtained by ML are presented for all the individual datasets.

### Morphological Statistical Analysis

The results of the morphological comparison of the different species are presented in Supplementary Figure S5. The values are mean of 50 measurements (±) SD, and significant differences according to Dunnett-t3′ multiple-range tests at *p* < 0.05 levels were indicated and followed by different letters.

### Taxonomy

Among the 178 strains obtained in this study, 12 species were identified. Nine of these species are new to science and are described as follows:

#### *Geosmithia luteobrunnea* R. Chang and X. Zhang, sp. nov.

MycoBank MB839256

Etymology: *luteobrunnea*, referring to the yellowish-brown appearance of the colony on MEA.

**FIGURE 2:**
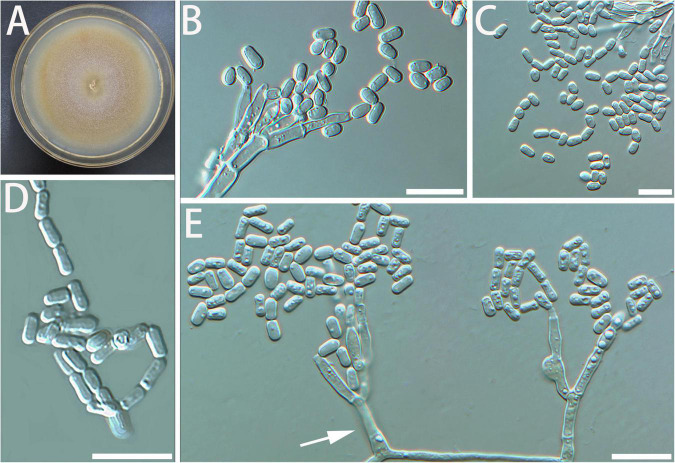
Morphological characteristics of *Geosmithia luteobrunnea* sp. nov. (SNM261 = CGMCC3.20252, SNM226, SNM287). **(A)** Eight-day-old culture on 2% malt extract agar. **(B–E)** Conidiophores and conidia. The stipe (indicated with arrows) is thick and short. Scale bars: 10 μm **(B–E)**.

Diagnosis: The stipe of *G. luteobrunnea* is slightly thicker and shorter than that in other species. *Geosmithia luteobrunnea* can grow at 5 and 35°C, even grows slowly at 37°C (Figure 2).

Type: China, Jiangxi Province, Ganzhou City, Longnan County, Jiulianshan National Nature Reserve (24°34′1′′ N, 114°30′ E), from the gallery of *Scolytus jiulianshanensis* on *Ulmus* sp., 5 May 2020, S. Lai, Y. Xu, S. Liao, Y. Wen and T. Li (HMAS 249919 – holotype, SNM261 = CGMCC3.20252 – ex-holotype culture).

Description: Sexual state not observed. Asexual state penicillium-like and (19.0–) 29.6–61.5 (–85.0)-μm long. *Conidiophores* borne mostly from aerial fungal hyphae, erect, determinate, solitary, sometimes funiculose, with all parts verrucose; base often consisting of a curved and atypically branched cell, stipe (6.4–) 11.3–40.1 (–78.4)-μm long, (1.5–) 1.7–3.2 (–6.0)-μm wide; penicillus, monoverticillate to terverticillate (penicilli of conidiophores on aerial funiculose mycelia are monoverticillate or biverticillate), symmetric or asymmetric, often irregularly branched, rami (1st branch) in whorls of 1–3, (4.1–) 5.2–7.0 (–8.7) × (1.2–) 1.7–2.5 (–3.2) μm, metulae (last branch) in whorls of 1 to 2, (4.0–) 4.9–6.5 (–7.6) × (1.4–) 1.8–2.3 (–2.6) μm; phialides in whorls of 1–3, cylindrical, without or with short cylindrical neck and smooth to verrucose walls, (4.2–) 5.1–7.5 (–10.2) × (1.1–) 1.5–2.3 (–2.7) μm. *Conidia* hyaline to subhyaline, smooth, narrowly cylindrical to ellipsoidal, (2.3–) 2.9–4.0 (–4.7) × (0.9–) 1.2–1.7 (–2.2) μm, produced in non-persistent conidial chains. Substrate conidia absent.

MEA, 8 days: Colony diameter 50–64 mm at 20°C, 58–78 mm at 25°C, and 44–70 mm at 30°C. The hyphae grow slowly at 5 and 35°C. After 8 days of culture, the colony diameter was 1.5–4 mm and 11–14 mm, respectively. The optimal temperature for growth was 25°C. Colonies at 25°C, 8 days, were oppressed, velutinous, or floccose with raised mycelial cords; colony margin smooth, filamentous, diffuse; aerial mycelium sparse; substrate mycelium sparse; conidiogenesis moderate; milky white to light yellow; reverse lighter brown; absence of exudate; no soluble pigment. When incubated at 35°C, colonies were raised, slightly depressed at the center, rugose, or irregularly furrowed; margin undulate somewhat erose; aerial mycelia sparse to moderate; substratum mycelia dense, forming a tough basal felt; the colony was darker and yellowish-brown; reverse brown; soluble pigment was brown. MEA, 37°C, 8 days, germinating only.

Host: *Liquidambar formosana*, *Liquidambar styraciflua*, *Ulmus* sp.

Beetle vectors: *Acanthotomicus suncei*, *Scolytus jiulianshanensis*.

Distribution: Currently only known from Jiangxi and Shanghai.

Notes: *Geosmithia luteobrunnea* and *G. radiata* are phylogenetically close to each other on ITS, TUB2, RPB2, TEF1-α trees, and combined alignment tree (Figure 1 and Supplementary Figures S1–S4). The colony morphology of *G. luteobrunnea* and *G. radiata* are also similar, but there are many differences among those two species. First of all, their sequences are different (Table 3). Then, under the microscope, the morphological differences between them are more obvious (Supplementary Figure S5). The spore of *G. radiata* is shorter than the other specie. The stipe of *G. radiata* is thicker than the other specie, and the stipe of *G. luteobrunnea* is slightly shorter than the other two species (Supplementary Figure S5). Moreover, their growths at different temperatures are also different (Table 4). *G. luteobrunnea* can grow at both temperatures, especially at 35°C, even grows slowly at 37°C. *Geosmithia radiata* only grows a little at 5°C and grows slowly at 35°C. The growth speed of *G. luteobrunnea* is faster than that of *G. radiata* (Table 4). *Geosmithia luteobrunnea* and *G. radiata* form a species group outstanding by cream to yellow or brown color of sporulation accompanied by the darker (brownish to rusty) shades of the substrate mycelium and colony reverse. This feature is shared also by the phylogenetically related *Geosmithia* sp. 11 (Kolařík et al., 2007) which is known from Europe and the Mediterranean (Kolařík et al., 2007, 2008) and seems to be diagnostic for the whole species group.

**TABLE 3:** Summary of the variability between species of the *Geosmithia luteobrunnea* species complex.

Species	ITS rDNA (555 bp)		TEF1-α (899 bp)		TUB2 (666 bp)		RPB2 (1066 bp)	
	*G. radiata*	*G. luteobrunnea*	*G. radiata*	*G. luteobrunnea*	*G. radiata*	*G. luteobrunnea*	*G. radiata*	*G. luteobrunnea*
*G. radiata*		5 (0.90%)		8–9 (0.89–1.0%)		4 (0.60%)		6 (0.56%)

**TABLE 4:** The colony diameter of *G. subfulva*, *G. bombycine*, *G. luteobrunnea*, *G. radiata*, *G. granulate*, and *G. pallida* species complex, *G. brevistipitata* and *G. pumila*, at different temperatures after 8 days on malt extract agar medium (unit: millimeter).

Species/T	5°C	20°C	25°C	30°C	35°C	37°C
*G. bombycina*	1	20–23	24–31	22–30	5–8	0
*G. brevistipitata*	2 to 3	20–30	23–34	8–12	0	0
*G. fusca*	1–6	21–26	25–36	26–32	1–11	≈0
*G. granulata*	<1	27–32	30–34	8–12	2–4	0
*G. luteobrunnea*	1–4	50–64	58–78	44–70	11–14	≈0
*G. pulverea*	0	23–29	30–37	31–36	1.5–4	0
*G. pumila*	7–10	25–29	25–33	22–26	≈0	0
*G. radiata*	1	50–58	59–69	49–60	1–4	0
*G. subfulva*	4–6	17–26	24–36	20–29	35	0

Additional cultures examined: China, Shanghai, from the gallery of *Acanthotomicus suncei* on *Liquidambar styraciflua*, April 2019, L. Gao (SNM226, SNM287).

#### *Geosmithia radiata* R. Chang and X. Zhang, sp. nov.

MycoBank MB839257

Etymology: *radiata*, referring to the radial appearance of the colony on MEA.

**FIGURE 3:**
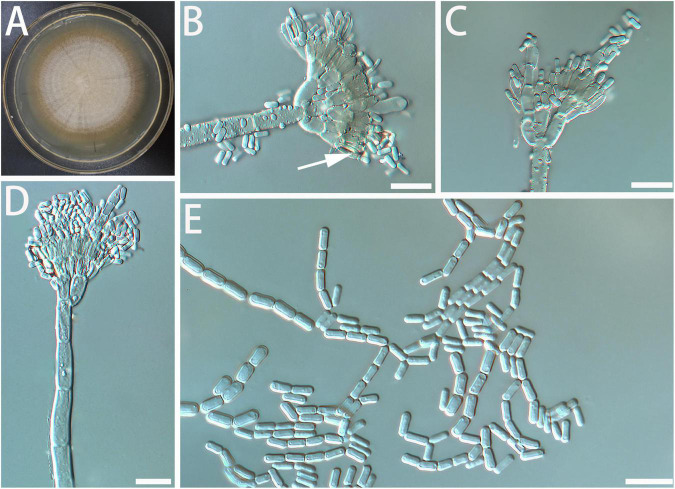
Morphological characteristics of *Geosmithia radiata* sp. nov. (SNM279 = CGMCC3.20253, SNM884). **(A)** Eight-day-old culture on 2% malt extract agar. **(B–E)** Conidiophores and conidia. The sporulation structure is coarse, and the phialides (indicated with arrows) are abundant and compact. Scale bars: 10 μm **(B–D)** and 20 μm **(E)**.

Diagnosis: The spore and the stipe of *G. radiata* are thicker than closely related species. *Geosmithia radiata* only grows a little at 5 and 35°C (Figure 3).

Type: China, Jiangxi Province, Ganzhou City, Longnan County (24°5′2.4′′ N, 114°47′2.4′′ E), from the gallery of *Acanthotomicus suncei* on *Liquidambar formosana*, 5 May 2020, S. Lai (HMAS 249920 – holotype, SNM279 = CGMCC3.20253 – ex-holotype culture).

Description: Sexual state not observed. Asexual state penicillium-like and (22.6–) 35.6–85.7 (–119.3)-μm long. *Conidiophores* borne from the substrate or aerial hyphae, sometimes arising laterally from another conidiophore, erect, determinate, solitary, with all parts verrucose; stipe commonly (7.3–) 18.4–63.6 (–115.8)-μm long, (1.6–) 2.1–3.8 (–5.9)-μm wide, penicillus, with walls thick, septate; penicillus terminal, monoverticillate, biverticillate, or terverticillate, mostly symmetrical, rami (1st branch) in whorls of 2 to 3, (4.2–) 5.2–7.8 (–10.6) × (1.3–) 2.1–3.5 (–4.8) μm; metulae (last branch) in whorls of 1 to 2, (2.6–) 3.9–5.8 (–7.3) × (1.3–) 1.7–2.6 (–3.3) μm. Phialides in whorls of 1–5, (3.9–) 4.6–6.2 (–7.7) × (1.5–) 1.9–2.8 (–3.9) μm, cylindrical, without or with short cylindrical neck and smooth to verrucose walls. *Conidia* cylindrical to ellipsoidal, smooth, hyaline to subhyaline, (2.2–) 2.5–3.2 (–4.0) × (0.9–) 1.1–1.5 (–1.8) μm, formed in non-persistent conidial chains. Substrate conidia absent.

MEA, 8 days: Colony diameter 50–58 mm at 20°C, 59–69 mm at 25°C, and 49–60 mm at 30°C. The hyphae grow slowly at 5 and 35°C. After 8 days of culture, the colony diameter was only 1 and 1–4 mm, respectively. The optimal temperature for growth is 25°C. Colonies at 25°C, 8 days, plane, slightly raised centrally, velutinous, with a slight overgrowth of aerial mycelium, with floccose and funiculose areas; substrate mycelium darker, aerial mycelium hyaline; sporulation moderate to heavy, pale cream; vegetative mycelium hyaline; reverse lighter yellow; soluble pigment and exudate absent. When incubated at 35°C, colonies were rising, slightly sunken in the center, furrowed, or irregularly fringed; the substratum hyphae were dense and formed a tough basal felt. The colony is dark and yellowish-brown. MEA, 37°C, 8 days: no growth.

Host: *Liquidambar formosana*, *Ulmus* sp.

Beetle vectors: *Acanthotomicus suncei*, *Scolytus jiulianshanensis.*

Distribution: Jiangxi.

Notes: See comparisons between *Geosmithia luteobrunnea*, *G. radiata* below the description of *G. luteobrunnea*.

Additional cultures examined: China, Jiangxi Province, Ganzhou City, Xunwu County (24°57′ N, 115°38′2′′ E), from the gallery of *Acanthotomicus suncei* on *Liquidambar formosana*, 5 May 2020 (SNM884).

#### *Geosmithia brevistipitata* R. Chang and X. Zhang, sp. nov.

MycoBank MB841503

Etymology: *brevistipitata*, referring to the short conidiophore stipe, a character distinguishing it from other members of the species complex.

**FIGURE 4:**
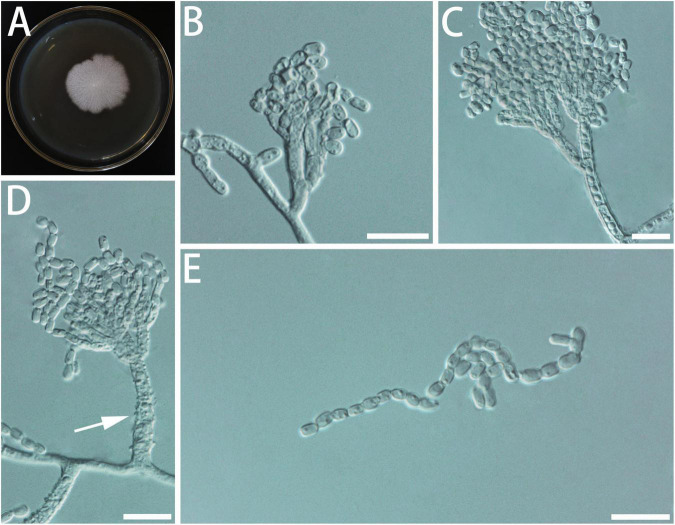
Morphological characteristics of *Geosmithia brevistipitata* sp. nov. (SNM1616 = CGMCC3.20627, SNM1610, SNM1611). **(A)** Eight-day-old culture on 2% malt extract agar. **(B–E)** Conidiophores and conidia. The stipe (indicated with arrows) is short and sometimes not smooth. Scale bars: 10 μm **(B–E)**.

Diagnosis: Isolates of *G. brevistipitata* formed a monophyletic clade on all the phylogenetic trees (Figure 4).

Type: China, Shandong Province, Linyi City, Tianfo scenic spot (35°5′ N, 118°2′ E), from the gallery of *Phloeosinus* cf. *hopehi* on *Cupressus funebris*, 8 August 2021, Y. Cao (HMAS 351566 - holotype, SNM1616 = CGMCC3.20627 – ex-holotype culture).

Description: Sexual state not observed. Asexual state penicillium-like and (9.5–) 15.5–42.3 (–77.9)-μm long. *Conidiophores* borne from the substrate or aerial hyphae, sometimes arising laterally from another conidiophore, erect, determinate, solitary, with all parts verrucose; stipe commonly (2.9–) 7.5–30.0 (–56.0) × (1.3–) 1.9–3.0 (–4.1) μm, penicillus, with walls thick, septate; penicillus terminal, monoverticillate or biverticillate, mostly symmetrical, metulae in whorls of 2–3, (4.6–) 6.3–9.1 (–11.2) × (1.8–) 2.0–2.7 (–3.2) μm. Phialides in whorls of 2–5, (3.2–) 5.0–8.7 (–11.4) × (1.3–) 1.7–2.4 (–2.8) μm, cylindrical, without or with short cylindrical neck and smooth to verrucose walls. Conidia cylindrical to ellipsoidal, smooth, hyaline to subhyaline, (2.2–) 2.4–3.1 (–3.8) × (1.2–) 1.5–1.9 (–2.2) μm, formed in non-persistent conidial chains. Substrate conidia absent.

MEA, 8 days: Colony diameter 24–30 mm at 20°C, 23–34 mm at 25°C, and 8–12 mm at 30°C. The hyphae grow slowly at 5°C. After 8 days of culture, the colony diameter was only 2–3 mm. No growth at 35°C. The optimal temperature for growth is 20–25°C. Colonies at 25°C, 8 days, plane, granular, with a slight growth of aerial mycelium; substrate mycelium white; reverse white; sporulation moderate white; soluble pigment and exudate absent. MEA, 37°C, 8 days: no growth.

Host: *Cupressus funebris.*

Beetle vectors: *Phloeosinus* cf. *hopehi.*

Distribution: Shandong.

Notes: Isolates of *G. brevistipitata* formed a monophyletic clade on both ITS, TUB2, TEF1-α, and RPB2 trees. Its closely related species differ on various trees, such as according to ITS tree, isolates of *G. brevistipitata* were closely related to *G. cnesini*, *G. xerotolerans*, *G. omnicola*, *G. ulmacea*, and *Geosmithia* sp. 12 (Supplementary Figure S5), but according to the TUB2 tree, isolates of *G. brevistipitata* were connected to other species, e.g., *G. microcorthyli* and *G. obscura* (Supplementary Figure S2). Among the other species described, it is outstanding by the combination of slow growth and white colony color and short stipe.

Additional cultures examined: China, Shandong Province, Linyi City, Tianfo scenic spot (118°2′ N, 35°5′ E), from the gallery of *Phloeosinus* cf. *hopehi* on *Cupressus funebris*, 8 August 2021, Y. Cao (SNM1610).

#### *Geosmithia granulata* R. Chang and X. Zhang, sp. nov.

MycoBank MB 840646

Etymology: *granulata*, referring to the granular appearance of the colony on MEA.

**FIGURE 5:**
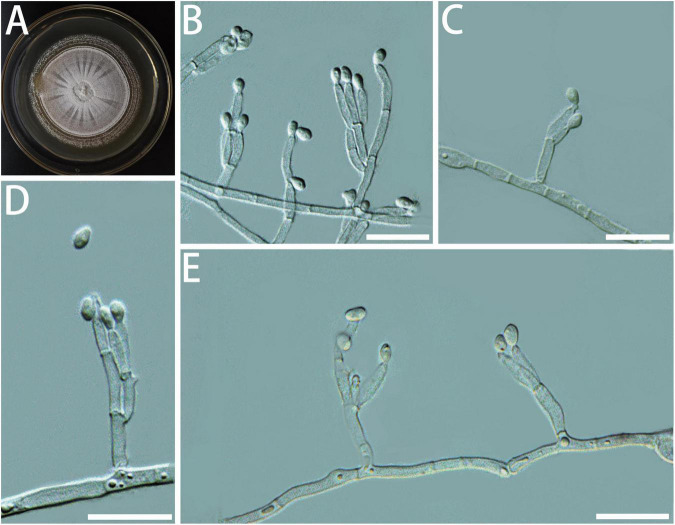
Morphological characteristics of *Geosmithia granulata* sp. nov. (SNM1015 = CGMCC3.20450, SNM1013). **(A)** Eight-day-old culture on 2% malt extract agar. **(B–E)** Conidiophores and conidia. Conidia hyaline, smooth, wide oval shape, like an egg. Scale bars: 10 μm **(B–E)**.

Diagnosis: The conidia of *G. granulata* are shorter than the closely related species (Figure 5).

Type: China, Yunnan Province, Xishuangbanna City, Xishuangbanna Botanical Garden (21°55′1′′ N, 101°16′1′′ E), from the gallery of *Sinoxylon* cf. *cucumella* on *Acacia pennata*, 1 May 2021, Y. Dong and Y. Li (HMAS 351568 - holotype, SNM1015 = CGMCC3.20450 – ex-holotype culture).

Description: Sexual state not observed. Asexual state penicillium-like, (9.6–) 11.6–26.0 (–50.6) μm in length. Conidiophores emerging from hyphae, smooth, septate; stipe (4.0–) 4.8–8.3 (–14.3) × (1.1–) 1.4–2.1 (–2.6) μm; penicilli typically longer than the stipe, terminal, monoverticillate, biverticillate, or terverticillate, symmetric or asymmetric, often irregularly branched, rarely more; metulae in whorls of 1–2, (5.2–) 5.7–8.1 (–11.3) × (1.0–) 1.3–1.7 (–2.0) μm; phialides in whorls of 1–4, smooth, (3.3–) 4.9–7.1 (–8.8) × (1.0–) 1.3–1.8 (–2.2) μm. Conidia hyaline, smooth, wide oval shape, like an egg, (1.5–) 1.8–2.2 (–2.5) × (0.8–) 1.0–1.4 (–1.8) μm. Conidia formed in long, non-persistent conidial chains. Substrate conidia absent.

MEA, 8 days: Colony diameter 27–32 mm at 20°C, 30–34 mm at 25°C, and 8–12 mm at 30°C. At 5°C: germinating only. At 35°C, the mycelia grew slowly. After 8 days of culture, the diameter of the colony was 2–4 mm. The optimal growth temperature is 20–25°C. At 25°C, 8 days: Colonies were flat and radiated, surface texture powdery; central hyphae slightly raised and wrinkled, conidiogenesis heavy; marginal colonies were similar to annual rings, slightly flocculent, hyphae were sparse, milky white, reverse creamyrice white; without exudate and insoluble pigment. MEA, 37°C, 8 days, no growth.

Host: *Acacia pennata*, *Hibiscus tiliaceus*, *Ulmus* sp.

Beetle vectors: *Sinoxylon* cf. *cucumella*, *Ernoporus japonicus*, *Scolytus semenovi.*

Distribution: Guangdong, Jiangsu, Yunnan.

Notes: According to the tree made by ITS and TEF1-α sequence, SNM1015 and SNM1013 were clustered with *Geosmithia* sp. 20 (Supplementary Figures S1, S3). TUB2 and RPB2 sequences of *Geosmithia* sp. 20 were not available on GenBank; therefore, *Geosmithia* sp. 20 was not included in TUB2 and RPB2 trees. These results suggested that our isolates and *Geosmithia* sp. 20 belonged to the same species, described as *G. granulata* sp. nov. This extends the geographical distribution of this species to the Mediterranean Basin (Kolařík et al., 2007) and western part of the United States (Kolařík et al., 2017) where it was found in association with many bark beetle species feeding on plants from the families Asteraceae, Fabaceae, Moraceae, Oleaceae, Ulmaceae (Mediterranean Basin), or Cupressaceae, Ulmaceae (Western United States).

Additional cultures examined: China, Yunnan Province, Xishuangbanna City, Xishuangbanna Botanical Garden (21°55′1′′ N, 101°16′1′′ E), from the gallery of *Sinoxylon* cf. *cucumella* on *Acacia pennata*, 1 May 2021, Y. Dong and Y. Li (SNM1013).

#### *Geosmithia subfulva* R. Chang and X. Zhang, sp. nov.

MycoBank MB 841505

Etymology: *subfulva*, referring to the beige appearance of the colony on MEA.

**FIGURE 6:**
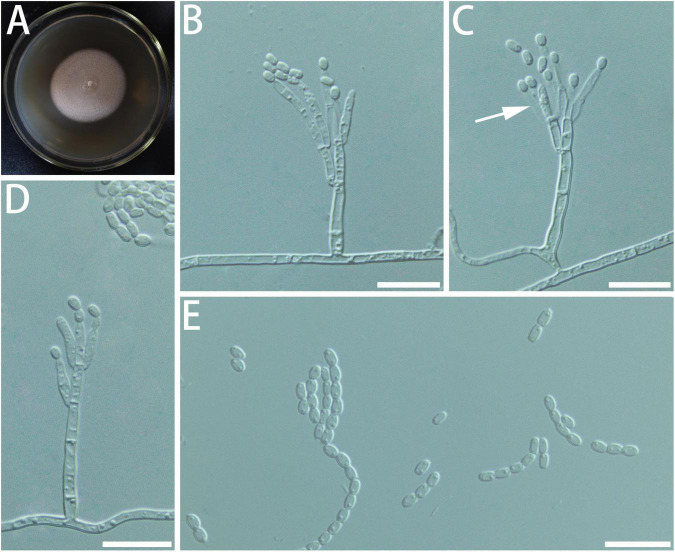
Morphological characteristics of *Geosmithia subfulva* sp. nov. (SNM1304 = CGMCC3.20579, SNM1298). **(A)** Eight-day-old culture on 2% malt extract agar. **(B–E)** Conidiophores and conidia. The metulae (indicated with arrows) branches are few and sparse. Scale bars: 10 μm **(B–E)**.

Diagnosis: Isolates of *G. subfulva* formed a monophyletic clade on all the phylogenetic trees (Figure 6).

Type: China, Guangdong Province, Zhuhai City (22°16′48′′ N, 113°30′28′′ E), from the gallery of *Ernoporus japonicus* in the twig of *Hibiscus tiliaceus*, 21 June 2021, W. Lin (HMAS 351569 - holotype, SNM1304 = CGMCC3.20579 – ex-holotype culture).

Description: Sexual state not observed. Asexual state penicillium-like and (13.3–) 21.0–43.5 (–62.5)-μm long. Conidiophores arising from substrate or aerial mycelium with all parts verrucose; stipe (5.3–) 9.3–26.4 (–36.6) × (0.9–) 1.5–2.2 (–3.1) μm; penicillus, biverticillate to quaterverticillate, symmetric or asymmetric, often irregularly branched, rarely more, rami (1st branch) in whorls of 1–2, (4.8–) 5.6–7.4 (–8.4) × (1.0–) 1.3–1.8 (–2.0) μm, metulae (last branch) in whorls of 1–3, (4.0–) 4.6–5.9 (–6.9) × (0.9–) 1.2–1.6 (–1.8) μm; phialides 1–3, cylindrical or ellipsoidal, without or with short cylindrical neck and smooth to verrucose walls, (3.6–) 4.8–6.9 (–10.0) × (0.8–) 1.1–1.4 (–1.6) μm. Conidia hyaline, smooth, wide oval shape, (1.1–) 1.5–2.2 (–2.2) × (1.0–) 1.1–1.5 (–1.7) μm. Conidia formed in long, non-persistent conidial chains. Substrate conidia absent.

MEA, 8 days: Colony diameter 17–26 mm at 20°C, 24–36 mm at 25°C, and 20–29 mm at 30°C. At 5 and 35°C, the mycelia grew slowly. After 8 days of culture, the colony diameter was 4–6 and 3–5 mm. The optimal growth temperature is 25°C. Colonies at 25°C, 8 days, plane with radial rows and slightly raised centrally, texture velutinous (powdery); beige to off-white; reverse milky white; soluble pigment and exudate absent. When incubated at 35°C, the colonies are the same as described above. MEA, 37°C, 8 days: no growth.

Host: *Hibiscus tiliaceus*, *Rhus chinensis.*

Beetle vectors: *Cryphalus kyotoensis*, *Ernoporus japonicus*, *Hypothenemus* sp. L636.

Distribution: Fujian, Guangdong, Shandong.

Notes: Isolates SNM1304 and SNM1298 formed a monophyletic clade on both ITS, TUB2, TEF1-α, and RPB2 trees (Supplementary Figures S1–S4). On the ITS tree, SNM1304 and SNM1298 were distinct from all other species. On the TUB2 tree, SNM1304 and SNM1298 are linked to *G. pulverea* but have no strong support. On TEF1-α and RPB2 trees, they are nested in a clade including not only *G. pulverea* but also several other species.

Additional cultures examined: China, Guangdong Province, Zhuhai City (22°16′48′′ N, 113°30′28′′ E), from the gallery of *Ernoporus japonicus* LW320 in the twig of *Hibiscus tiliaceus*, 21 June 2021, W. Lin (SNM1298).

#### *Geosmithia pulverea* R. Chang and X. Zhang, sp. nov.

MycoBank MB839259

Etymology: *pulverea*, powdery in Latin. On MEA medium, *G. pulverea* has powdery sporulation.

**FIGURE 7:**
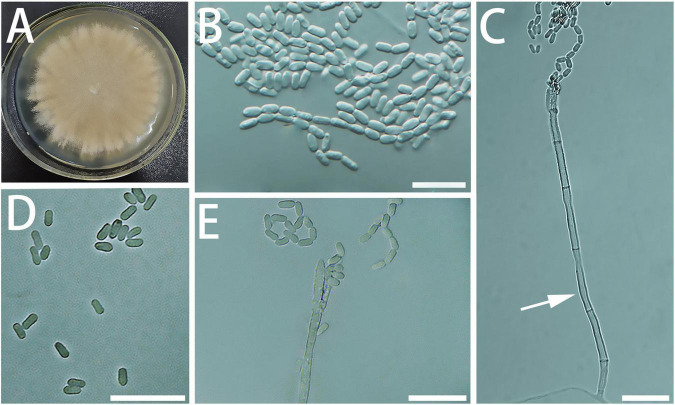
Morphological characteristics of *Geosmithia pulverea* sp. nov. (SNM885 = CGMCC3.20255, SNM270, SNM888). **(A)** Eight-day-old culture on 2% malt extract agar. **(B–E)** Conidiophores and conidia. The stipe (indicated with arrows) are slender and abundant with spores. Scale bars: 10 μm **(B–E)**.

Diagnosis: *Geosmithia pulverea* produces long spore chains, while its closely related species does not (Figure 7).

Type: China, Guangdong Province, Shenzhen City (21°55′12″ N, 101°16′12″ E), from the gallery of *Dinoderus* sp. L489 in the vine of *Gnetum luofuense*, 12 April 2018, Y. Li (HMAS 249922 – holotype, SNM885 = CGMCC3.20255 – ex-holotype culture).

Description: Sexual state not observed. Asexual state penicillium-like and (17.5-) 30.9–84.3 (-120.1)-μm long. *Conidiophores* arising from substrate or aerial mycelium with all parts verrucose; base often consisting of curved and atypically branched cell; stipe (16.2–) 32.7–85.7 (–153.9) × (1.9–) 2.5–3.7 (–4.7) μm; penicillus, biverticillate to quaterverticillate, symmetric or asymmetric, often irregularly branched, rarely more, rami (1st branch) in whorls of 2–4, (8.2–) 10.2–14.4 (–18.9) × (2.2–) 2.5–3.3 (–3.9) μm, metulae (last branch) in whorls of 2–3, (6.3–) 7.5–10.9 (–15.8) × (1.8–) 2.1–2.8 (–3.5) μm; phialides 1–3, cylindrical or ellipsoidal, without or with short cylindrical neck and smooth to verrucose walls, (5.3–) 7.0–9.6 (–12.3) × (1.5–) 1.8–2.5 (–3.0) μm. Conidia hyaline, smooth, narrowly cylindrical to ellipsoidal, (2.1–) 2.5–3.4 (–5.1) × (1.1–) 1.2–1.6 (–2.0) μm. Conidia formed in long, non-persistent conidial chains. Substrate conidia absent.

MEA, 8 days: Colony diameter 23–29 mm at 20°C, 30–37 mm at 25°C, and 31–36 mm at 30°C. No growth at 5°C. At 35°C, the mycelia grew slowly. After 8 days of culture, the colony diameter was 1.5–4 mm, with a yellow soluble pigment. The optimal growth temperature is 25–30°C. Colonies at 25°C, 8 days, plane with radial rows and slightly raised centrally, texture velutinous (powdery); sporulation abundant, light brownish yellow to buff; reverse yellowish to slightly avellaneous brown; soluble pigment and exudate absent. When incubated at 35°C, the colonies are the same as described above. MEA, 37°C, 8 days: no growth.

Host: *Acacia pennata*, *Gnetum luofuense*, *Liquidambar formosana*, *L. styraciflua*, *Choerospondias axillaris*, Lauraceae, *Eriobotrya japonica*, *Rhus chinensis*, *Ulmus* spp.

Beetle vectors: *Sinoxylon* cf. *cucumella*, *Acanthotomicus suncei*, *Crossotarsus emancipatus*, *Dinoderus* sp. L489, *Microperus* sp. L589, *Phloeosinus* sp., *Scolytus semenovi*, *Scolytus jiulianshanensis*, *Cryphalus kyotoensis*, *Cryphalus eriobotryae.*

Distribution: Fujian, Guangdong, Guangxi, Hunan, Jiangxi, Yunnan, Shandong, Shanghai.

Notes: *Geosmithia pulverea* colony was powdery and brown-yellow. One of the most obvious features is the long spore chain. According to the tree made by ITS sequence, SNM888, SNM885, and SNM248 were clustered with *Geosmithia* sp. 3, and SNM886, SNM887, and SNM270 were clustered with *Geosmithia* sp. 23 (Supplementary Figure S1). However, in the trees with TUB2, TEF1-α, and RPB2, these strains did not have a clear subclassification (Supplementary Figures S2–S4). It was consequently recognized, using multigene phylogeny, together with *Geosmithia* sp. 23, as a well-defined phylogenetic species inside the *G. pallida* species complex (Huang et al., 2017; Kolařík et al., 2017). The colony of *G. pulverea* was very similar to *Geosmithia* sp. 3 on MEA, but *Geosmithia* sp. 3 was darker and wrinkled (Kolařík et al., 2004). *Geosmithia pulverea* seems to have a smaller stipe size, but other features fit the morphology of *Geosmithia* sp. 3 (Kolařík et al., 2004). In this study, we are providing a formal description for the Chinese strains related to *Geosmithia* sp. 3 and sp. 23 which are known to be distributed over various bark beetle hosts in Temperate Europe in the case of *Geosmithia* sp. 3 (Kolařík et al., 2004, 2008; Strzałka et al., 2021) or seems to have a global distribution and many bark beetle hosts across Temperate Europe (Strzałka et al., 2021), the Mediterranean Basin (Kolařík et al., 2007), Northern America (Huang et al., 2017, 2019; Kolařík et al., 2017), and Seychelles (Kolařík et al., 2017). Further study is needed to assess the taxonomic relationships between *G. pulverea*, *Geosmithia* sp. 3, and *Geosmithia* sp. 23.

Additional cultures examined: China, Guangxi Province, Shangsi City, Shiwandashan Mt. (21°54′12′′ N, 107°54′14′′ E), from the body surface of *Crossotarsus emancipates*, 27 March 2018, Y. Li (SNM887).

China, Hunan Province, Changsha City, Yuelushan Mt. (28°10′56′′ N, 112°55′41′′ E), from the gallery of *Microperus* sp. L589 on the trunk of *Choerospondias axillaris*, 15 July 2019, Y. Li (SNM888).

#### *Geosmithia fusca* R. Chang and X. Zhang, sp. nov.

MycoBank MB841506

Etymology: *fusca*, referring to the brown appearance of the colony on MEA.

**FIGURE 8:**
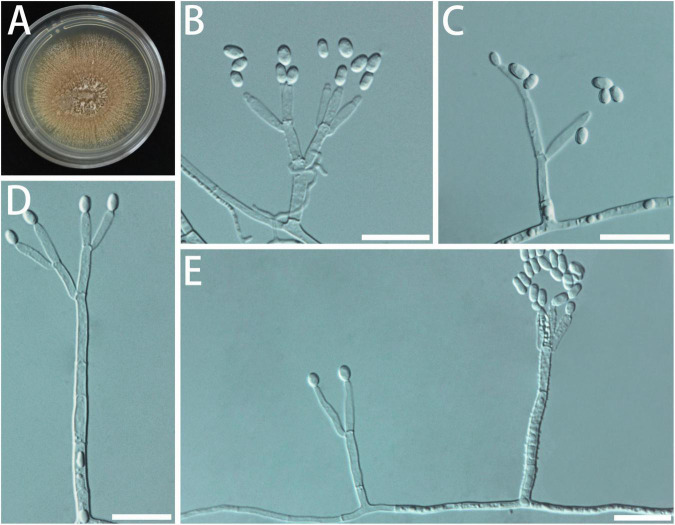
Morphological characteristics of *Geosmithia fusca* sp. nov. (SNM1578 = CGMCC3.20626, SNM1577). **(A)** Eight-day-old culture on 2% malt extract agar. **(B–E)** Conidiophores and conidia. Conidia hyaline, smooth, wide oval shape. Scale bars: 10 μm **(B–E)**.

Diagnosis: The difference with closely related species *G. cucumellae* is reflected in such a way that the conidia of *G. fusca* are smooth and do not produce long spore chains (Figure 8).

Type: China, Guangdong Province, Zhuhai City, Agricultural Science Research Center (22°18′9′′ N, 113°31′40′′ E), from the gallery of *Xylocis tortilicornis* on *Phyllanthus emblica*, 6 July 2021, W. Lin (HMAS 351570 - holotype, SNM1578 = CGMCC3.20626 – ex-holotype culture).

Description: Sexual state not observed. Asexual state penicillium-like and (16.3–) 20.2–55.8 (–94.3)-μm long. Conidiophores variable in shape and size, emerging from a surface mycelium, with all segments smooth or minutely verrucose to distinctly verrucose, septate, stipe (8.6–) 10.1–38.5 (–70.1) × (1.2–) 1.5–2.1 (–2.6) μm; penicilli typically shorter than the stipe, terminal, monoverticillate or biverticillate, symmetric or asymmetric, irregularly branched; metulae in whorls of 2–3, (4.9–) 6.0–8.3 (–9.9) × (1.1–) 1.3–1.8 (–2.1) μm; phialides in whorls of 1–3, smooth, (4.6–) 5.8–8.1 (–9.4) × (1.0–) 1.3–1.7 (–1.9) μm. Conidia cylindrical to ellipsoid, (1.5–) 2.0–2.7 (–3.4) × (0.9–) 1.1–1.7 (–1.7) μm. Conidia formed in long, non-persistent conidial chains. Substrate conidia absent.

MEA, 8 days: Colony diameter 21–26 mm at 20°C, 25–36 mm at 25°C, and 26–32 mm at 30°C. At 5 and 35°C, the mycelia grew slowly. After 8 days of culture, the colony diameter was 1–3 and 7–11 mm, respectively. The optimal growth temperature is 25–30°C. At 25°C, 8 days: Colonies flat with radial rows, surface texture powdery; sporulation abundant, brown; central hyphae were raised and white flocculent; reverse yellowish to brown; without exudate and insoluble pigment. MEA, 37°C, 8 days: germinating only.

Host: *Hibiscus tiliaceus*, *Phyllanthus emblica*, *Acacia pennata.*

Beetle vectors: *Ernoporus japonicus*, *Xylocis tortilicornis*, *Sinoxylon* cf. *cucumella.*

Distribution: Guangdong, Yannan.

Notes: In the phylogenetic tree, SNM1012, SNM1067 and SNM1577, SNM1578 formed very close separate branches (Figure 1 and Supplementary Figures S1–S4), but combined with morphological analysis, it was found that SNM1012, SNM1067 and SNM1577, SNM1578 had no significant difference except for a small difference in spore length (Supplementary Figure S5). So, they are described as the same species.

Additional cultures examined: China, Guangdong Province, Zhuhai City, Agricultural Science Research Center (22.3025 N, 113.5277 E), from the gallery of *Xylocis tortilicornis* LW319 on *Phyllanthus emblica*, 6 July 2021, W. Lin (SNM1577).

China, Yunnan Province, Xishuangbanna City, Xishuangbanna Botanical Garden (21°55′1′′ N, 101°16′1′′ E), from the gallery of *Sinoxylon* cf. *cucumella* on the trunk of *Acacia pennata*, 1 May, 2021, Y. Dong and Y. Li (SNM1012, SNM1167).

#### *Geosmithia pumila* R. Chang and X. Zhang, sp. nov.

MycoBank MB841507

Etymology: *pumila*, referring to the tree host of *Ulmus pumila* where this species has been isolated.

**FIGURE 9:**
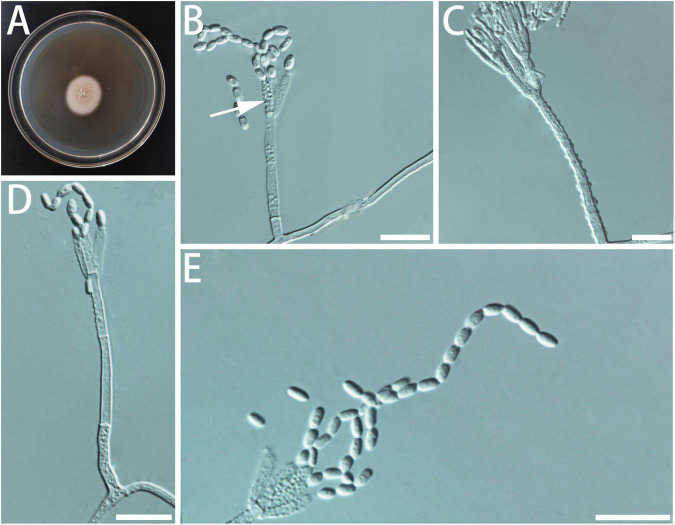
Morphological characteristics of *Geosmithia pumila* sp. nov. (SNM1653 = CGMCC3.20630, SNM1657). **(A)** Eight-day-old culture on 2% malt extract agar. **(B–E)** Conidiophores and conidia. Most phialides (indicated with arrows) are not smooth. Scale bars: 10 μm **(B–E)**.

Diagnosis: Isolates of *G. pumila* formed a monophyletic clade on all the phylogenetic trees (Figure 9).

Type: China, Jiangsu Province, Nanjing City, Nanjing Forestry University (32°3′36′′ N, 118°48′36′′ E), from the gallery of *Scolytus semenovi* in the branch of *Ulmus* sp., 25 August 2021, S. Lai (HMAS 351571 - holotype, SNM1653 = CGMCC3.20630 – ex-holotype culture).

Description: Sexual state not observed. Asexual state penicillium-like and (12.9–) 35.9–72.7 (–109.4)-μm long. Conidiophores arising from substrate or aerial mycelium with all parts verrucose; stipe (9.9–) 19.7–51.5 (–77.9) × (1.2–) 1.4–2.2 (-2.6) μm; penicillus, monoverticillate or biverticillate, mostly monoverticillate, symmetric or asymmetric, often irregularly branched, rarely more, metulae in whorls of 2 to 3, (5.1–) 6.3–8.9 (–10.5) × (1.1–) 1.4–2.0 (–2.3) μm; phialides 1–3, smooth to verrucose walls, (5.0–) 5.7–7.3 (–8.5) × (1.1–) 1.2–1.6 (–1.8) μm. Conidia hyaline, smooth, ellipsoidal, (1.5–) 1.9–2.5 (–2.9) × (0.9–) 1.1–1.5 (–1.9) μm. Conidia formed in long, non-persistent conidial chains. Substrate conidia absent.

MEA, 8 days: Colony diameter 25–29 mm at 20°C, 25–33 mm at 25°C, and 22–26 mm at 30°C. At 35°C: germinating only. At 5°C, the mycelia grew slowly. After 8 days of culture, the colony diameter was 7–10 mm. The optimal growth temperature is 20–25°C. Colonies at 25°C, 8 days, plane with radial rows, texture velutinous (powdery), slightly funiculus centrally; sporulation medium, light yellow to rice white; reverse milk-white; soluble pigment and exudate absent. MEA, 37°C, 8 days: no growth.

Host: *Ulmus* sp.

Beetle vectors: *Scolytus semenovi.*

Distribution: Jiangsu.

Notes: Based on ITS, TUB2, TEF1-α, and RPB2 trees (Supplementary Figures S1–S4), SNM1653 and SNM1657 were grouped with *Geosmithia* sp. 2. Therefore, we considered that SNM1653, SNM1657, and *Geosmithia* sp. 2 were the same species. This extends the geographical range to Europe, the Mediterranean Basin (Kolařík et al., 2007, 2008), the whole United States (Huang et al., 2017, 2019; Kolařík et al., 2017), Peru (Kolařík et al., 2004), and South Africa (Machingambi et al., 2014), which is reported to be in association with a large number of insect and tree hosts.

Additional cultures examined: China, Jiangsu Province, Nanjing City, Nanjing Forestry University (32°3′36′′ N, 118°48′36′′ E), from the gallery of *Scolytus semenovi* in the branch of *Ulmus* sp., 25 August 2021, S. Lai (SNM1657).

#### *Geosmithia bombycina* R. Chang and X. Zhang, sp. nov.

MycoBank MB 840535

Etymology: *bombycina*, referring to the cotton appearance of the colony on MEA.

**FIGURE 10:**
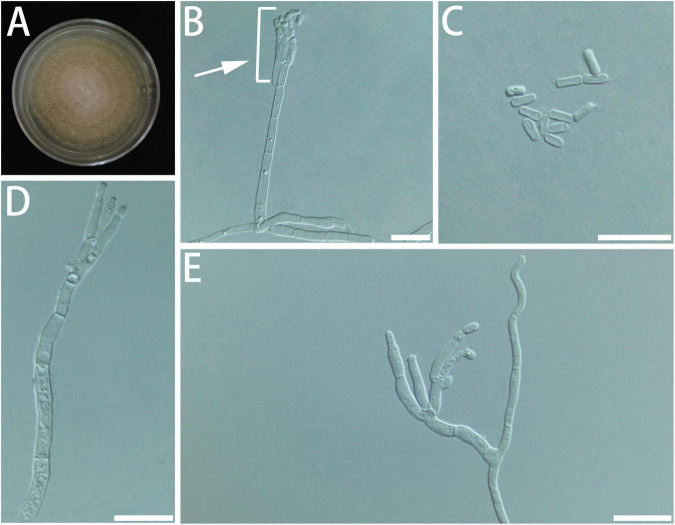
Morphological characteristics of *Geosmithia bombycina* sp. nov. (SNM933 = CGMCC3.20578, SNM934). **(A)** Eight-day-old culture on 2% malt extract agar. **(B–E)** Conidiophores and conidia. The penicilli (indicated with arrows) are typically shorter than the stipe, terminal, monoverticillate, biverticillate or terverticillate, symmetric or asymmetric. Scale bars: 10 μm **(B–E)**.

Diagnosis: Isolates of *G. bombycina* formed a monophyletic clade on all the phylogenetic trees (Figure 10).

Type: China, Fujian Province, Fuqing City (25°71′ N, 119°15′ E), from the gallery of *Cryphalus eriobotryae* on *Eriobotrya japonica*, 8 April 2021, Y. Li (HMAS 350284 – holotype, SNM933 = CGMCC3.20578 – ex-holotype culture).

Description: Sexual state not observed. Asexual state penicillium-like, (14.0–) 20.2–41.0 (–62.6) μm in length. Conidiophores emerging from hyphae, smooth, septate; stipe (5.4–) 9.4–30.0 (–47.5) × (0.9–) 1.4–2.0 (–2.4) μm; penicilli typically shorter than the stipe, terminal, monoverticillate, biverticillate or terverticillate, symmetric or asymmetric, often irregularly branched, rarely more; metulae in whorls of 1–2, (5.1–) 5.9–8.3 (–10.5) × (1.0–) 1.2–1.7 (–2.1) μm; phialides in whorls of 2–4, smooth, (4.9–) 5.8–9.4 (–12.6) × (0.9–) 1.3–1.7 (–2.0) μm. Conidia hyaline, smooth, narrow, and oval, (2.1–) 2.4–3.3 (–4.1) × (0.8–) 0.9–1.3 (–1.5) μm, produced in non-persistent chains. Substrate conidia absent.

MEA, 8 days: Colony diameter 20–23 mm at 20°C, 24–31 mm at 25°C, and 22–30 mm at 30°C. The hyphae grow slowly at 5 and 35°C. After 8 days of culture, the colony diameter was less than 1 and 5–8 mm, respectively. The optimal temperature for growth was 25–30°C. At 25°C, 8 days: The colonies were flat, like annual rings; central hyphae were raised and white flocculent; filamentous, diffuse, basal mycelium sparse; conidiogenesis moderate, milk-white; reverse creamy white; no exudate and insoluble pigment. When incubated at 35°C, the colonies grew, and the mycelia were sparse and snowflake-shaped, with no soluble pigment. MEA, 37°C, 8 days, no growth.

Host: *Eriobotrya japonica.*

Beetle vectors: *Cryphalus eriobotryae.*

Distribution: Currently only known from Fujian.

Notes: According to ITS, TUB2, and TEF1-α trees (Supplementary Figures S1–S4), SNM933 and SNM934 formed a monophyletic clade and nested with *Geosmithia* sp. 22, *Geosmithia* sp. 24, *G. longistipitata*, *G. pazoutovae*, and *G. fagi*. The RPB2 sequences for those species were not available on GenBank. Therefore, SNM933 and SNM934 formed a distinct clade that was far away from all the known species on the RPB2 tree.

Additional cultures examined: China, Fujian Province, Fuqing City (25°71′ N, 119°15′ E), from the gallery of *Cryphalus eriobotryae* on *Eriobotrya japonica*, 8 April 2021, Y. Li (SNM934).

## Discussion

This is the first relatively comprehensive study of *Geosmithia* species associated with bark beetle in China. The samples were collected from 9 provinces, 12 tree hosts, and 12 bark and ambrosia beetles. A total of 178 strains of *Geosmithia* were isolated in this study. The analyses of ITS, RBP2, TUB2, and TEF1-α showed that those isolates were separated into 12 taxa, with three strains previously described, *G. xerotolerans*, *G. putterillii*, and *G. pallida*, and the other nine were novel species, described as *G. luteobrunnea*, *G. radiata*, *G. brevistipitata*, *G. bombycina*, *G. granulata* (*Geosmithia* sp. 20), *G. subfulva*, *G. pulverea* (*Geosmithia* sp. 3 and *Geosmithia* sp. 23), *G. fusca*, and *G. pumila* in this study. Those species were isolated from larvae, frass, and wood dust in the beetle galleries of dying, stressed, or weakened broad-leaf and conifer tree hosts, such as *Liquidambar* spp., *Ulmus* sp., and *Cupressus* sp.

The dominant species obtained in this study were *G. luteobrunnea* and *G. pulverea*, with 39 and 33 strains, respectively (Table 1). The reason for their abundance in our dataset is the fact that our study focused on sampling from *Altinginaceae*. Two species, *G. putterillii* and *G. radiata*, have only been isolated in Jiangxi (Table 1). The samples collected from Guangxi and Hunan only yielded *G. pulverea*.

*Geosmithia putterillii* was isolated from bark beetles feeding on plants from the family of Rosaceae (Kolařík et al., 2008) and Lauraceae in Europe (Kolařík et al., 2004) and on various families of angiosperms and gymnosperms in the Western United States (Kolařík et al., 2017). The type strain was isolated from timber in New Zealand (Pitt, 1979). In this study, *G. putterillii* was isolated from the gallery of *Phloeosinus* sp. on Lauraceae log (Jiangxi). This study is the first report of *G. putterillii* in China. It is becoming clear that *G. putterillii* is widely distributed globally, across many beetle hosts.

Another known species collected in this study is *G. pallida*, originally isolated from cotton yarn and soil (Kolařík et al., 2004). Later, it was found to be associated with beetles, such as ambrosia beetle *Xylosandrus compactus* (Vannini et al., 2017), and plants such as *Brucea mollis* (Deka and Jha, 2018). *G. pallida* was previously reported to induce dieback poisoning on coast live oak (*Quercus agrifolia*) by Lynch et al. (2014). Later, it was proved that the identification was incorrect, and the causal agent of this disease was confirmed to be *Geosmithia* sp. 41 (Kolařík et al., 2017). Two isolates were obtained from the gallery of *Sinoxylon* cf. *cucumella* on *Acacia pennata* in this study, which is the first report of *G. pallida* in China.

Most of *G. luteobrunnea* were isolated from the galleries of *A. suncei* (Table 1). *Acanthotomicus suncei* was recorded on *Liquidambar* in Fujian, Jiangsu, Jiangxi, Zhejiang, and Shanghai, China (Li et al., 2021). The hosts of this beetle were limited to sweet gum trees, such as *L. styraciflua* and *L. formosana*. The beetle was recorded as an agent of great damage to the imported American sweetgum *L. styraciflua* in Shanghai and neighboring Jiangsu Province (Gao and Cognato, 2018). The role of the fungus in this outbreak and the tree pathology remain uninvestigated, although the authors of this paper noted small lesions around the beetle galleries. The other five isolates were isolated from the galleries of *S. jiulianshanensis* on *Ulmus* sp., which suggests that *G. luteobrunnea* might colonize a wide range of tree hosts.

*Geosmithia radiata* was only isolated in samples from Jiangxi Province, from two plant families: Altinginaceae and Ulmaceae (Table 1). The colony of *G. radiata* is similar to *G. luteobrunnea* in morphology, but the difference can be seen in the micromorphology (Supplementary Figure S5). In addition, *G. luteobrunnea* can grow faster at 35°C, while *G. radiata* grows slower, and *G. luteobrunnea* could grow at 35°C, but *G. radiata* could not (Table 4).

*Geosmithia brevistipitata* and *G. xelotolerans* were isolated from the gallery of *Phloeosinus* cf. *hopehi*. This is not the first time that *Geosmithia* species were isolated from the gallery of *Phloeosinus* species. According to previous reports, *G. flava*, *G. longdonii*, *G. putterillii*, *G. Lavandula*, *etc*., are all related to *Phloeosinus* (Kolařík et al., 2017). It is now more certain that *Phloeosinus* and *Geosmithia* are closely related. *Geosmithia xelotolerans* is cosmopolite, known from the Mediterranean on many bark beetle species infecting Fabaceae, Moraceae, Oleaceae (Kolařík et al., 2007), in Western US on Cupressaceae, Pinaceae Fagaceae, Rosaceae (Kolařík et al., 2017), and in Eastern US on Cupressaceae, Fagaceae (Huang et al., 2017, 2019), and wall of the wall (Spain, Crous et al., 2018). Our study expanded the distribution range of *G. xelotolerans*.

*Geosmithia bombycina* was isolated from the gallery of *C. eriobotryae* on *E. japonica*. *Cryphalus eriobotryae* is one of the beetle pests that infest loquat (Zheng et al., 2019). This is the first study about the fungal association of this beetle.

*Geosmithia granulata* was isolated from the gallery of *Sinoxylon* cf. *cucumella* on *Acacia pennata*, *Ernoporus japonicus* on *Hibiscus tiliaceus*, and *Scolytus semenovi* on *Ulmus* sp. in this study. It was reported that it could be vectored by different beetle species which infested several plant hosts (Kolařík et al., 2007). In this study, we expanded the range of its beetle vectors and tree host.

*Geosmithia pulverea* is a species closely related to *Geosmithia* sp. 3 and *Geosmithia* sp. 23, which are known from various bark beetle hosts in Europe, United States, and Seychelles (Kolařík et al., 2007, 2008, 2017; Huang et al., 2017, 2019). Further study is needed to clarify the evolutionary relationship among these three lineages. In this study, we isolated *G. pulverea* from *Aca. gracilipes*, *Alt. gracilipes*, *E. japonica*, *Gne. luofuense*, *L. formosana, L. styraciflua*, *Rhus chinensis*, and *Ulmus* sp. (Table 1), which suggested that this species could colonize a very wide variety of plant hosts. It is also the most widely distributed species, isolated from Guangdong, Guangxi, Hunan, Jiangsu, Jiangxi, Shandong, and Shanghai (Table 1) and vectored by several beetle species, such as *S. jiulianshanensis*, *A. suncei*, *C. emancipatus*, *C. kyotoensis*, *Dinoderus* sp., *Microperus* sp., and *Phloeosinus* sp. (Table 1). Moreover, the abundance of *Geosmithia* species associated with *A. suncei* in the current study was also consistent with the frequent occurrence in Shanghai and Jiangxi (Gao et al., 2021).

In addition to *G. pallida*, *Geosmithia pulverea*, and *Geosmithia fusca* are the species found in the *G. pallida* species complex in this study. Only eight isolates of *G. fusca* were obtained from the gallery of *Sinoxylon* cf. *cucumella* on *Acacia pennata*. Two isolates of *G. pallida*, eight isolates of *G. pulverea*, and two isolates of *G. granulata* were also obtained from this beetle. Information about this beetle was very limited. As far as we know, it was found on Wendlandia tinctoria and distributed in the Himalayan mountain area and Burma, Thailand, Laos, and Vietnam (Liu, 2010; Liu and Beaver, 2018; Borowski, 2021). This is the first report in China, and this is the first study on its fungal associations.

## Conclusion

This study does not provide sufficient data to determine the structure of the *Geosmithia* community in China, as was inferred in Europe and United States after a significantly greater sampling effort (Kolařík et al., 2007, 2008, 2017; Kolařík and Jankowiak, 2013; Jankowiak et al., 2014; Huang et al., 2017, 2019). Fungal communities are regulated by several factors, including geographic location, host tree species, and bark beetle vectors. Further sampling is needed to understand the determinants (Veselská et al., 2019). It is clear, however, that the diversity of China’s subcortical fungi is substantial. Fungal communities associated with trees need to be further investigated because many currently unknown species may cause plant diseases.

## Data Availability Statement

The datasets presented in this study can be found in online repositories. The names of the repository/repositories and accession number(s) can be found in the article/Supplementary Material.

## Author Contributions

RC, MD, and YL designed the research. YL, HS, and GZ collected the samples. XZ, RC, and YL isolated and purified the fungal cultures. XZ, RC, and XJ completed the data acquisition, analyses, and interpretation. XZ and RC completed the writing of the manuscript. MK, JH, and YL revised the text, taxonomy, and phylogeny. All authors approved the manuscript.

## Author Disclaimer

This publication may not necessarily express the views of APHIS.

## Conflict of Interest

The authors declare that the research was conducted in the absence of any commercial or financial relationships that could be construed as a potential conflict of interest.

## Publisher’s Note

All claims expressed in this article are solely those of the authors and do not necessarily represent those of their affiliated organizations, or those of the publisher, the editors and the reviewers. Any product that may be evaluated in this article, or claim that may be made by its manufacturer, is not guaranteed or endorsed by the publisher.

## Footnotes

1
http://tree.bio.ed.ac.uk/software/tracer

2
https://www.treebase.org/

3
https://imagej.net/

4
https://www.ibm.com/analytics/spss-statistics-software

## Abbreviations

BI: Bayesian inference
ITS: nuclear ribosomal internal transcribed spacer
TEF1- α: translation elongation factor 1- α
TUB2: β-tubulin
ML: maximum likelihood
PCR: polymerase chain reaction
CGMCC: China General Microbiological Culture Collection Center
HMAS, Herbarium Mycologicum: Academiae Sinicae
TCD: thousand cankers disease.
