# Supplementary material for: Geosmithia Species Associated With Bark Beetles From China, With the Description of Nine New Species
Source: Front Microbiol. 2022 Mar 14;13:820402. doi: 10.3389/fmicb.2022.820402 (PMC8964297; doi:10.3389/fmicb.2022.820402)
Supplement: Supplementary file 1 [file Data_Sheet_1.PDF]

Supplementary Material

1 Supplementary Figures and Tables

1.1 Supplementary Figures

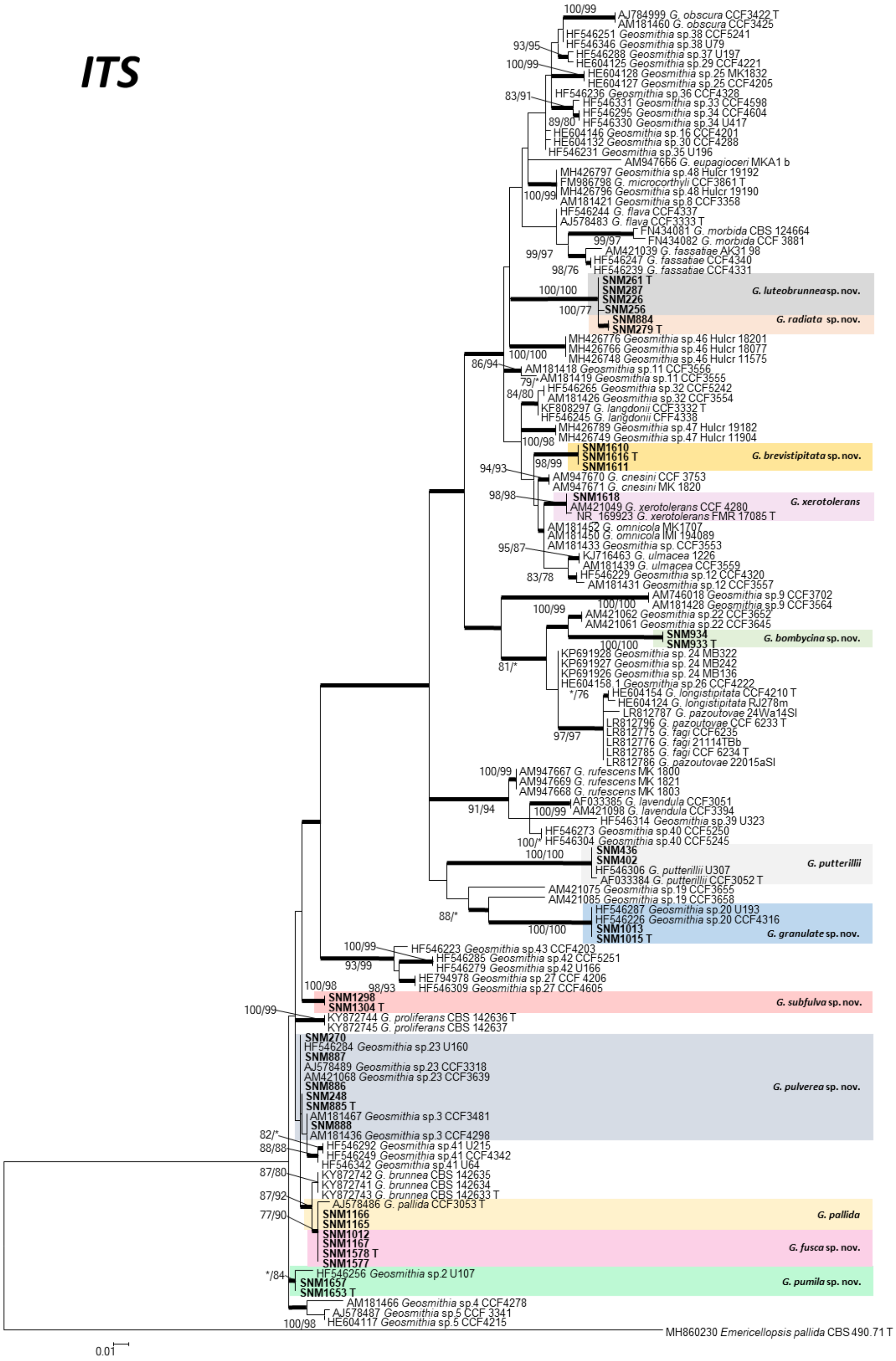

Supplementary Figure 1. ML tree of *Geosmithia* generated from the ITS sequence data. Sequences generated from this study are printed in bold. Bold branches indicate posterior probability values ⩾ 0.9. Bootstrap values of ML/MP ⩾ 75% are recorded at the nodes. T = ex-type isolates

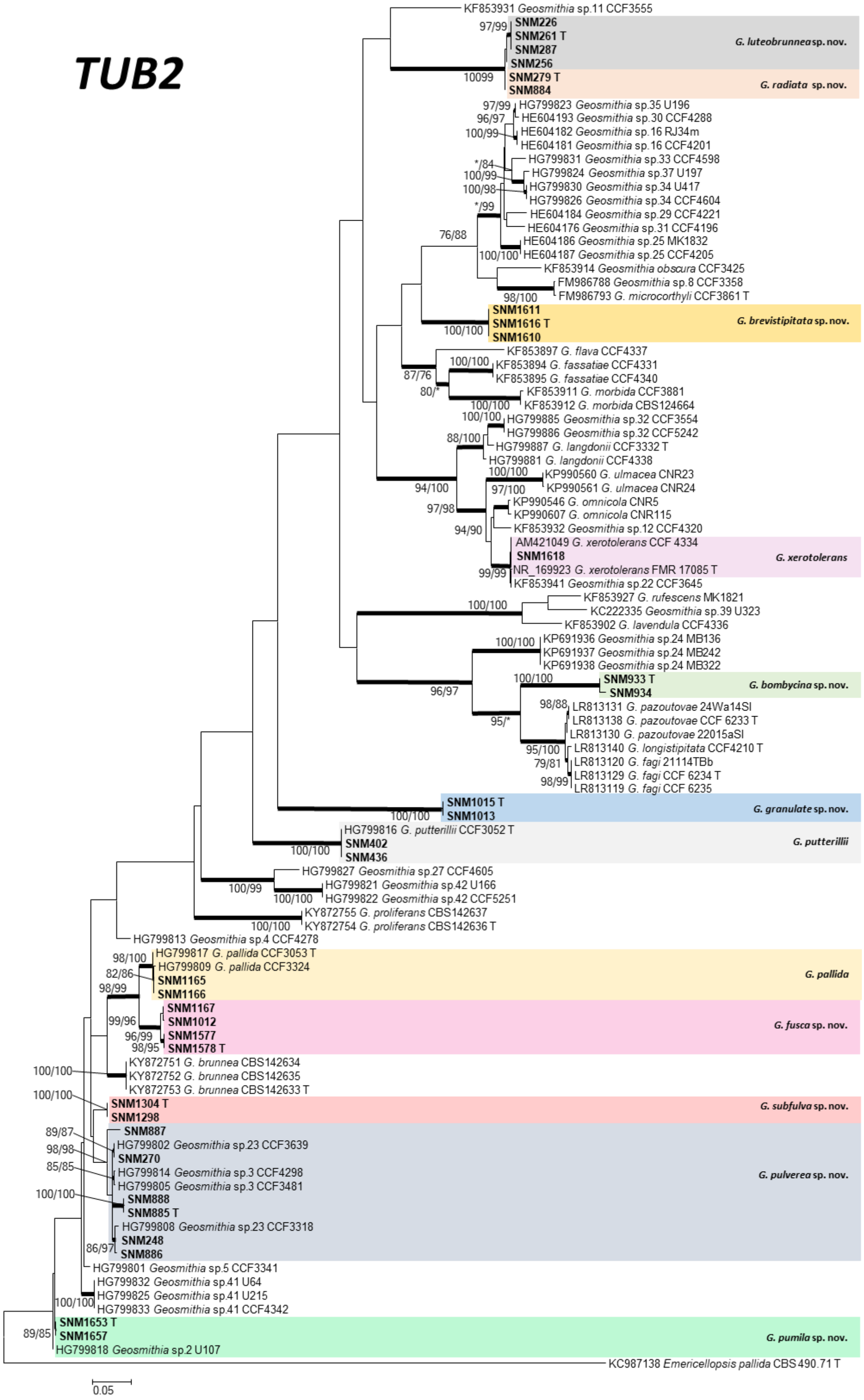

Supplementary Figure 2. ML tree of *Geosmithia* generated from the TUB2 sequence data. Sequences generated from this study are printed in bold. Bold branches indicate posterior probability values ≥ 0.9. Bootstrap values of ML/MP ≥ 75% are recorded at the nodes. T = ex-type isolates

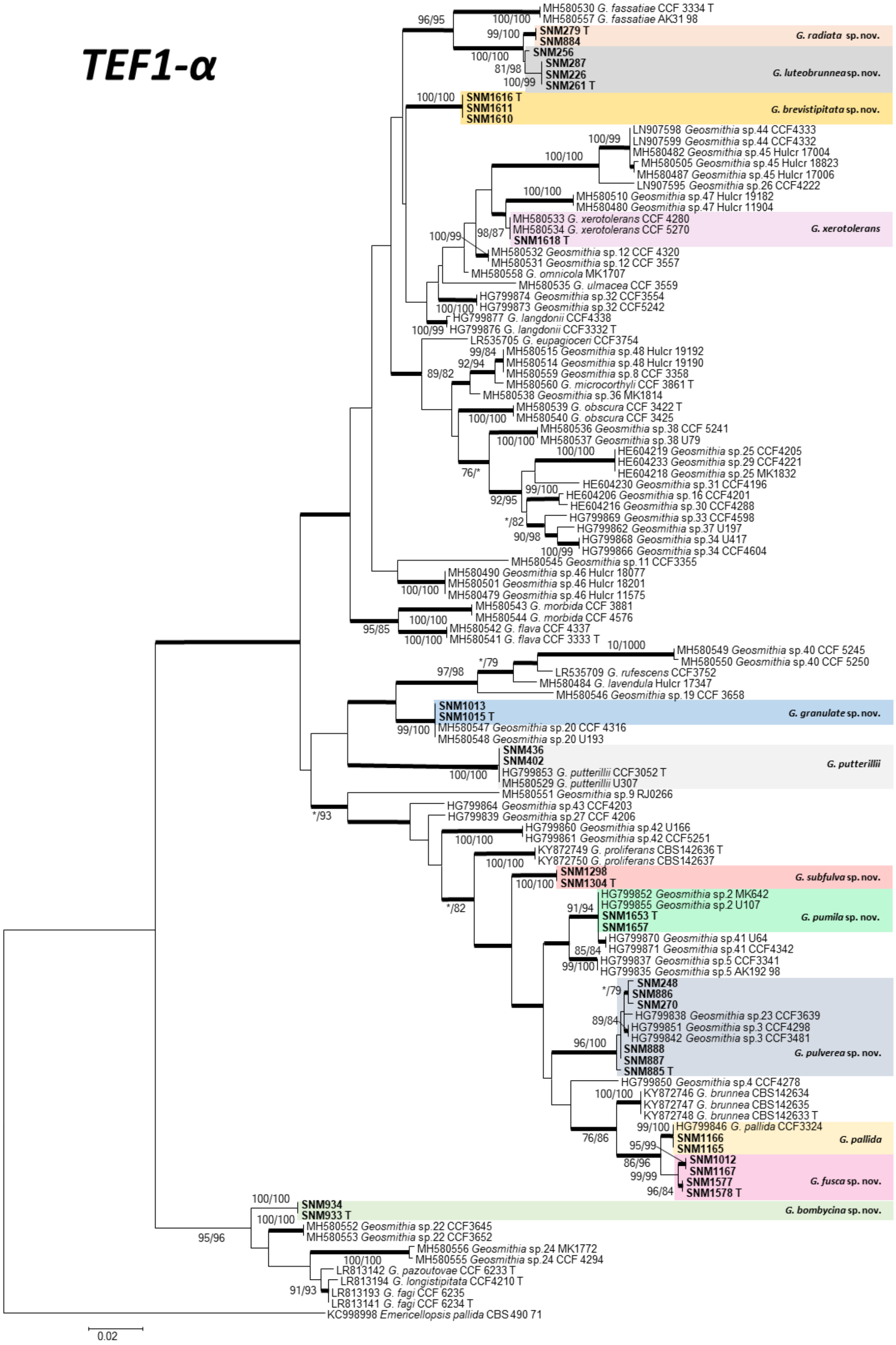

Supplementary Figure 3. ML tree of *Geosmithia* generated from the TEF1-α sequence data. Sequences generated from this study are printed in bold. Bold branches indicate posterior probability values ≥ 0.9. Bootstrap values of ML/MP ≥ 75% are recorded at the nodes. T = ex-type isolates

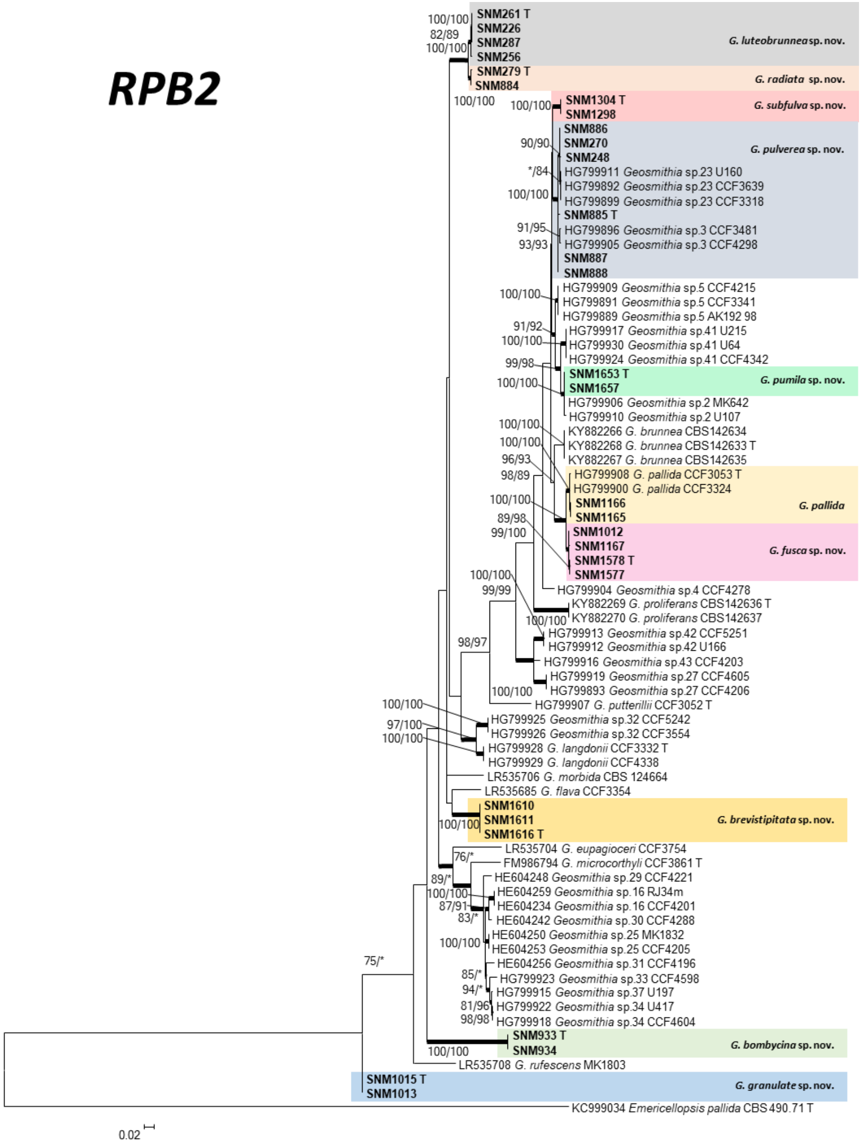

Supplementary Figure 4. ML tree of *Geosmithia* generated from the RPB2 sequence data. Sequences generated from this study are printed in bold. Bold branches indicate posterior probability values ≥ 0.9. Bootstrap values of ML/MP ≥ 75% are recorded at the nodes. T = ex-type isolates

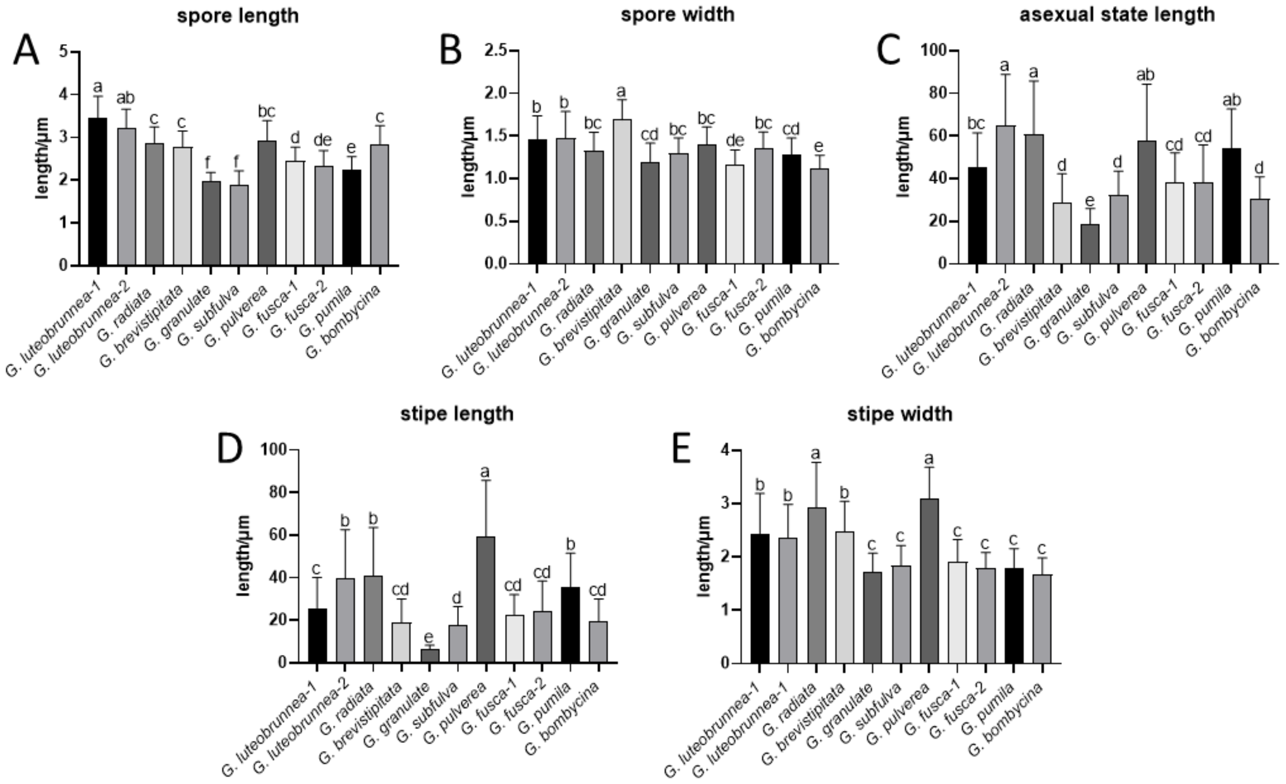

Supplementary Figure 5. The morphological comparison of the different species. Values are mean of 50 measurements (±) SD, and significant differences according to Dunnett-t3' multiple range tests at $p < 0.05$ levels were indicated and followed by different letters. **A.** Spore length; **B.** Spore width; **C.** Asexual state length; **D.** Stipe length; **E.** Stipe width

1.2 Supplementary Table

Supplementary Table 1. The result of the ITS marker BLAST on NCBI GenBank.

Group	Isolation no	Scientific Name	Per. ident
1	SNM261	*Geosmithia morbida*	95.5-96.5%
	SNM318		
	SNM319		
	SNM320		
	SNM321		
	SNM210		
	SNM226		
	SNM201		
	SNM285		
	SNM286		
	SNM280		
	…		
2	SNM1616	*Geosmithia omnicola*	98.0-98.5%
	SNM1600		
	SNM1601		
	SNM1602		
	SNM1603		
	SNM1605		
	SNM1606		
	SNM1607		
	SNM1608		
	SNM1609		
	SNM1610		
	SNM1611		
	SNM1612		
	SNM1615		
	SNM1617		
	SNM1619		
	SNM1621		
	SNM1662		
3	SNM1618	*Geosmithia xerotolerans*=*Geosmithia* sp. 21	99.2-100%
4	SNM934	*Geosmithia* sp. CCF3652	97.33%
	SNM933		
5	SNM1015	*Geosmithia* sp. 20	100%
	SNM1013		
	SNM1280		
	SNM1281		
	SNM1282		
	SNM1573		
	1547-1568(22)		
	SNM1654		
	SNM1659		
6	SNM402	*G. putterillii*	100%
	SNM436		
	SNM414		
	SNM415		
	SNM416		
	SNM417		
7	SNM1165	*G. pallida*	99.8-100%
	SNM1166		
	SNM1012		
	SNM1167		
	SNM1173		
	SNM1577		
	SNM1578		
	…		
8	SNM1304	*Geosmithia* sp. 23	99-100%
	SNM1298		
	SNM1583		
	SNM885		
	SNM886		
	SNM887		
	SNM888		
	SNM270		
	SNM248		

9	…<br>SNM1653<br>SNM1657	*Geosmithia* sp. 2	99.61%
